# Sp1 induced gene TIMP1 is related to immune cell infiltration in glioblastoma

**DOI:** 10.1038/s41598-022-14751-4

**Published:** 2022-07-01

**Authors:** Lu Liu, Shuyao Yang, Kefeng Lin, Xiaoman Yu, Jiaqi Meng, Chao Ma, Zheng Wu, Yuchao Hao, Ning Chen, Qi Ge, Wenli Gao, Xiang Wang, Eric W.-F. Lam, Lin Zhang, Fangcheng Li, Bilian Jin, Di Jin

**Affiliations:** 1 Institute of Cancer Stem Cell, Cancer Center, Dalian Medical University, Dalian, 116044 Liaoning People’s Republic of China; 2 Department of Neurosurgery, Guangzhou Women and Children’s Medical Center, Guangzhou, 510623 Guangdong People’s Republic of China; 3 State Key Laboratory of Oncology in South China, Collaborative Innovation Center for Cancer Medicine, Sun Yat-Sen University Cancer Center, Guangzhou, 510060 Guangdong People’s Republic of China

**Keywords:** Biomarkers, Predictive markers, Prognostic markers, Cancer, Cancer microenvironment, CNS cancer, Tumour biomarkers

## Abstract

Tumor immune microenvironment exerts a profound effect on the population of infiltrating immune cells. Tissue inhibitor of matrix metalloproteinase 1 (TIMP1) is frequently overexpressed in a variety of cells, particularly during inflammation and tissue injury. However, its function in cancer and immunity remains enigmatic. In this study, we find that TIMP1 is substantially up-regulated during tumorigenesis through analyzing cancer bioinformatics databases, which is further confirmed by IHC tissue microarrays of clinical samples. The TIMP1 level is significantly increased in lymphocytes infiltrating the tumors and correlated with cancer progression, particularly in GBM. Notably, we find that the transcriptional factor Sp1 binds to the promoter of TIMP1 and triggers its expression in GBM. Together, our findings suggest that the Sp1-TIMP1 axis can be a potent biomarker for evaluating immune cell infiltration at the tumor sites and therefore, the malignant progression of GBM.

## Introduction

Glioma is a primary central nervous system tumor that arises from glial cells. Under the WHO classification in 2016, gliomas can be divided into grades II, III, and IV (glioblastoma, GBM) by the extent of aggressiveness^1^. While the treatments have been developed in the past few decades, the median survival and prognosis are still unsatisfactory^2,3^. Recently, studies using next-generation sequencing have revealed that numerous genetic alterations can occur during brain carcinogenesis^4–6^. Several reports have discovered genomic alterations and differentially expressed genes (DEGs) as biomarkers for cancer progression of glioblastoma^6–10^. However, the underlying mechanisms of tumor biomarkers remain unknown. Hence, it should be promising if further studies attempt to focus on identifying novel glioma biomarkers and their mechanism.

Tumor immune microenvironment (TIME) consists of the immune components, including the immune cells, adjacent blood vessels, fibroblasts, signaling molecules, and the extracellular matrix, that surround and interact with the tumor. It has been reported that TIME bidirectionally affects the development of cancers^11–14^. Tumor-infiltrating immune cells play an essential part in cancer patient’s prognosis and response to systemic therapies, such as immunotherapy^13,15,16^. Normal brain tissues exist the blood-brain barrier (BBB) to protect neuron and glial cells against the influence of immunity and inflammation^17^. The structure and function of BBB could be weakened during the period of brain tumor growth and peripheral immune cells are recruited to the tumor to compose the TIME, which promotes the progression of tumor^18^. Specifically, immune cells could be guided by brain tumor cells to inhibit antitumor immune response and mediate the chemotherapy resistance in tumors^19,20^. Therefore, identifying novel molecular markers that correlate with these dysfunctional T cells remains crucial for designing promising and effective treatment strategies of glioblastoma.

The family of TIMP1 is composed of four members, TIMP1–4. TIMP1 is the main functional TIMP and is secreted and functions in the soluble form^21,22^. Numerous researches have indicated significantly high expression of TIMP1 in diverse cancer types^23–30^. The meta-analysis used in some studies evaluated the prognostic value of TIMP1 and found that plasma TIMP1 serves as an independent prognostic marker in some cancers^31–35^. There are also significant correlations between circulating TIMP1 and the TNM stages of gastric cancer, as well as its metastasis to distal organs^23^. In pancreatic cancer, a higher TIMP1 expression level correlates with worse reaction to therapies and liver metastasis^26^. TIMP1 has also been recognized as a biomarker for colorectal cancer^31,36^, breast cancer^35^, melanoma^24^ and papillary thyroid carcinoma^37^. However, hitherto, analysis of the prognostic value of TIMP1 has been limited to small sample size studies, and the relationship between TIMP1 expression and the changes in TIME is still unclear. Sp1 is an essential transcription factor that combines with GC-rich sequences, which are relevant to many target genes^38,39^. Sp families (Sp1–4) are transcription factors and play central roles in many cellular processes, including cell cycles and development^40–42^. It has been found that Sp1 is often highly expressed in most tumors, including gastric, pancreatic, lung, brain (glioma) and thyroid cancer ^43–47^. In glioma, Sp1 promotes proliferation and invasion of glioma cells via upregulating oncogenes, such as ADAM17 and MDK^48,49^. In concordance, Sp1 is overexpressed in TMZ-resistant glioma cells, and inhibition of Sp1 restores the anticancer effects of TMZ^50^. Besides its effects on tumor cells, Sp1 is also correlated with inflammation and immune cell infiltration^51^. Furthermore, several analyses also indicated that Sp1 is associated with adverse immunological changes in brain glioma and small cell lung cancer^52,53^.

In this study, we analyzed cancer databases and identified TIMP1 and six other genes to be potential markers for driving cancer progression in GBM patients. Consistently, analysis of the expression level of TIMP1 showed that it is correlated with negative patient survival and immune infiltration in glioblastoma and other cancer types. In agreement, IHC analysis of clinical sample arrays confirmed our findings. Moreover, we demonstrated Sp1 interact with the TIMP1 promoter to upregulate its expression in glioma cells. Taken together, our results identify and demonstrate that the Sp1-TIMP1 axis is a prognostic and immune infiltration biomarker for glioblastoma.

## Results

### Identification of prognostic biomarkers in patients with glioblastoma

**Figure 1:**
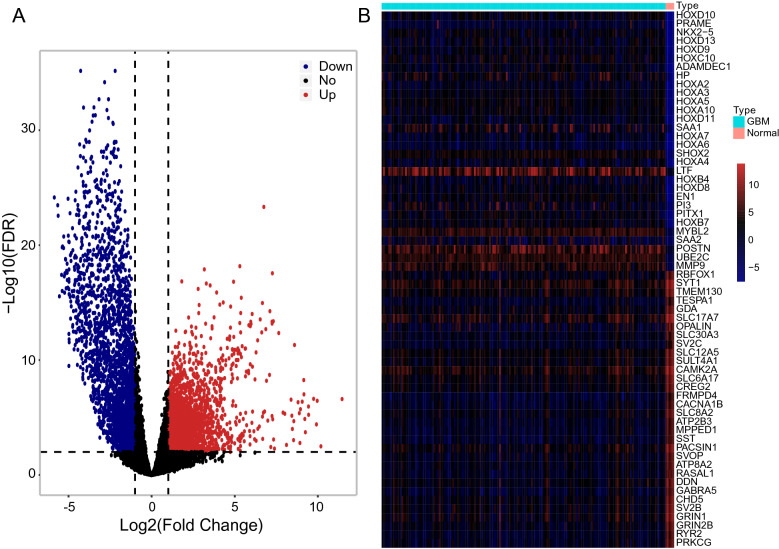
Differential expression of protein-coding genes in patients with glioblastoma. (**A**) Volcanic maps for differentially expressed genes. The x-axis specifies the log2 value of fold change (FC) and the y-axis specifies the negative log10 value of FDR (Adjusted P-value). Black dotted vertical and horizontal lines reflect the filtering criteria (FC =  ± 2 and FDR = 0.01). Red dots represent 2413 significantly upregulated genes. Blue dots represent 2435 significantly downregulated genes. Black dots represent nondifferentially expressed genes. (**B**) Heat maps of the top 30 most upregulated and downregulated genes in normal and GBM patients.

**Figure 2:**
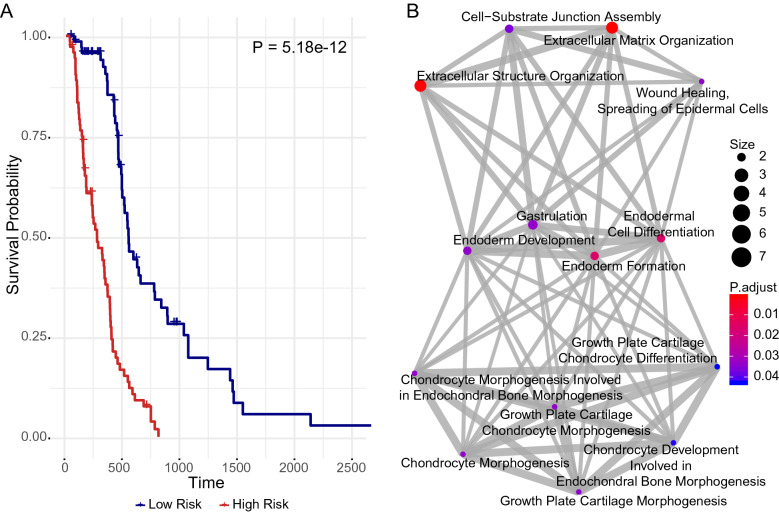
The up-regulated potential prognostic genes are correlated with prognosis and the processes of cell biology. (**A**) Survival curves of patients with Glioblastoma. The high-risk group indicates the high-risk score and the low-risk group indicates the low-risk score. The groups between low and high-risk scores were stratified according to the median expression level of the risk score. Data were analyzed by log-rank test.The X-axis indicates time in days. (**B**) the results of Go analysis about these 33 genes.

In order to identify the potential prognostic biomarkers, we retrieved the gene expression profiles from glioblastoma patients in TCGA. After excluding the patients who died within 30 days and protein-coding genes with less than ten counts in at least 75% of the total subjects, a total of 14,801 genes in 169 tumorous and five control subjects were analyzed using edgeR. We found that the expression of 2413 genes was upregulated, whereas 2435 genes were downregulated (Fig. 1A and Table S1). The top 30 most upregulated or downregulated genes were identified (Fig. 1B). Further analysis using the univariate factor cox regression model revealed 33 differentially expressed genes, which derived from 2413 upregulated genes, as potential prognostic biomarkers (Fig. S1C). Notably, LASSO regression was used to investigate the prognosis value of these potential biomarkers and we identified a risk score model (Fig.S1D-E). Indeed, when we separated these patients into high-risk and low-risk groups according to their risk score, a significantly different survival curve was observed (Fig. 2A). The gene enrichment analysis revealed that these 33 genes significantly contribute to cellular growth and development (Fig. 2B).

### Identification of prognostic biomarkers correlating with immune infiltration

**Figure 3:**
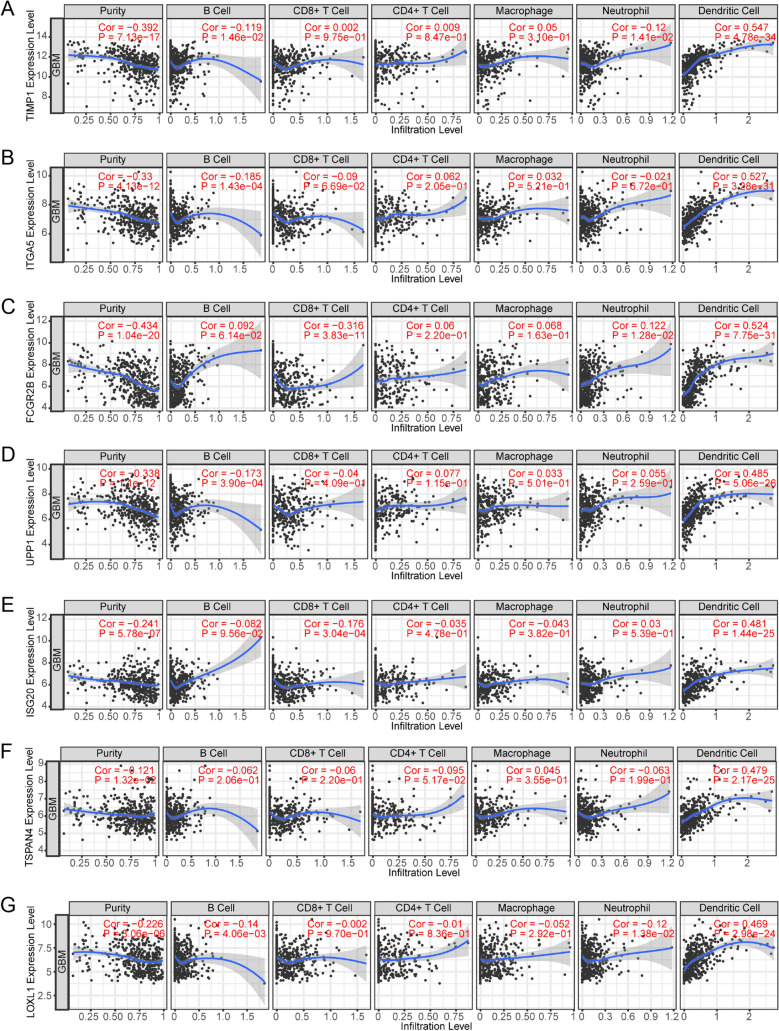
The 7 genes are potential biomarkers correlated with immune infiltration in GBM. TIMER analysis of the correlation betwenn immune infiltration and prognostic biomarkers (**A**) TIMP1, (**B**) ITGA5, (**C**) FCGR2B, (**D**) UPP1, (**E**) ISG20, (**F**) TSPAN4, and (**G**) LOXL1. Immune infiltration markers include purity, B cell, CD8+ T cell, CD4+ T cell, macrophage, neutrophil, and dendritic cell.

TIMER was utilized to investigate the relationships of the levels of these 33 genes in glioblastoma with the recruitment of immune cells, including B cells, CD8+ T cells, CD4+ T cells, macrophages, neutrophils, and dendritic cells. We found that TIMP1, ITGA5, FCGR2B, UPP1, ISG20, TSPAN4, and LOXL1 are potential biomarkers correlated with the immune infiltration events in patients with glioblastoma (Fig. 3 and Table 1). In all cases, the gene expression is significantly negatively related to tumor purity. TIMP1, ITGA5, UPP1, ISG20, TSPAN4, and LOXL1 have a significant positive correlation with infiltrating levels of dendritic cells, and FCGR2B has a significant positive correlation with infiltrating levels of neutrophils and dendritic cells.

**Table 1:** TIMER analysis of the correlation between immune infiltration and prognostic biomarkers.

Gene	Cancer	Variable	Partial.cor	p
TIMP1	GBM	Purity	−0.39238	7.13E−17
TIMP1	GBM	B cell	−0.11935	0.014622
TIMP1	GBM	CD8 + T cell	0.001509	0.975466
TIMP1	GBM	CD4 + T cell	0.00949	0.846605
TIMP1	GBM	Macrophage	0.049792	0.30983
TIMP1	GBM	Neutrophil	−0.11997	0.014113
TIMP1	GBM	Dendritic cell	0.547388	4.78E−34
ITGA5	GBM	Purity	−0.33012	4.13E−12
ITGA5	GBM	B cell	−0.18499	0.000143
ITGA5	GBM	CD8 + T cell	−0.08971	0.066889
ITGA5	GBM	CD4 + T cell	0.062059	0.205437
ITGA5	GBM	Macrophage	0.03151	0.520573
ITGA5	GBM	Neutrophil	−0.02077	0.671969
ITGA5	GBM	Dendritic cell	0.526705	3.28E−31
FCGR2B	GBM	Purity	−0.43435	1.04E−20
FCGR2B	GBM	B cell	0.091564	0.061434
FCGR2B	GBM	CD8 + T cell	−0.31596	3.83E−11
FCGR2B	GBM	CD4 + T cell	0.060154	0.21972
FCGR2B	GBM	Macrophage	0.068432	0.16255
FCGR2B	GBM	Neutrophil	0.121656	0.012808
FCGR2B	GBM	Dendritic cell	0.523878	7.75E−31
UPP1	GBM	Purity	−0.33842	1.10E−12
UPP1	GBM	B cell	−0.17269	0.00039
UPP1	GBM	CD8 + T cell	−0.04045	0.409469
UPP1	GBM	CD4 + T cell	0.077171	0.115168
UPP1	GBM	Macrophage	0.033028	0.50068
UPP1	GBM	Neutrophil	0.055288	0.259384
UPP1	GBM	Dendritic cell	0.484777	5.06E−26
ISG20	GBM	Purity	−0.2413	5.78E−07
ISG20	GBM	B cell	−0.08161	0.095635
ISG20	GBM	CD8 + T cell	−0.17582	0.000304
ISG20	GBM	CD4 + T cell	−0.03484	0.477514
ISG20	GBM	Macrophage	−0.04288	0.381863
ISG20	GBM	Neutrophil	0.030144	0.538825
ISG20	GBM	Dendritic cell	0.4808	1.44E−25
LOXL1	GBM	Purity	−0.2257	3.06E−06
LOXL1	GBM	B cell	−0.14027	0.004061
LOXL1	GBM	CD8 + T cell	−0.00183	0.970167
LOXL1	GBM	CD4 + T cell	−0.01016	0.835948
LOXL1	GBM	Macrophage	−0.05161	0.292494
LOXL1	GBM	Neutrophil	−0.12036	0.0138
LOXL1	GBM	Dendritic cell	0.469029	2.98E−24
TSPAN4	GBM	Purity	−0.12094	0.013236
TSPAN4	GBM	B cell	−0.06203	0.205676
TSPAN4	GBM	CD8 + T cell	−0.06016	0.219708
TSPAN4	GBM	CD4 + T cell	−0.09522	0.051731
TSPAN4	GBM	Macrophage	0.045384	0.354671
TSPAN4	GBM	Neutrophil	−0.06295	0.198958
TSPAN4	GBM	Dendritic cell	0.479248	2.17E−25

**Figure 4:**
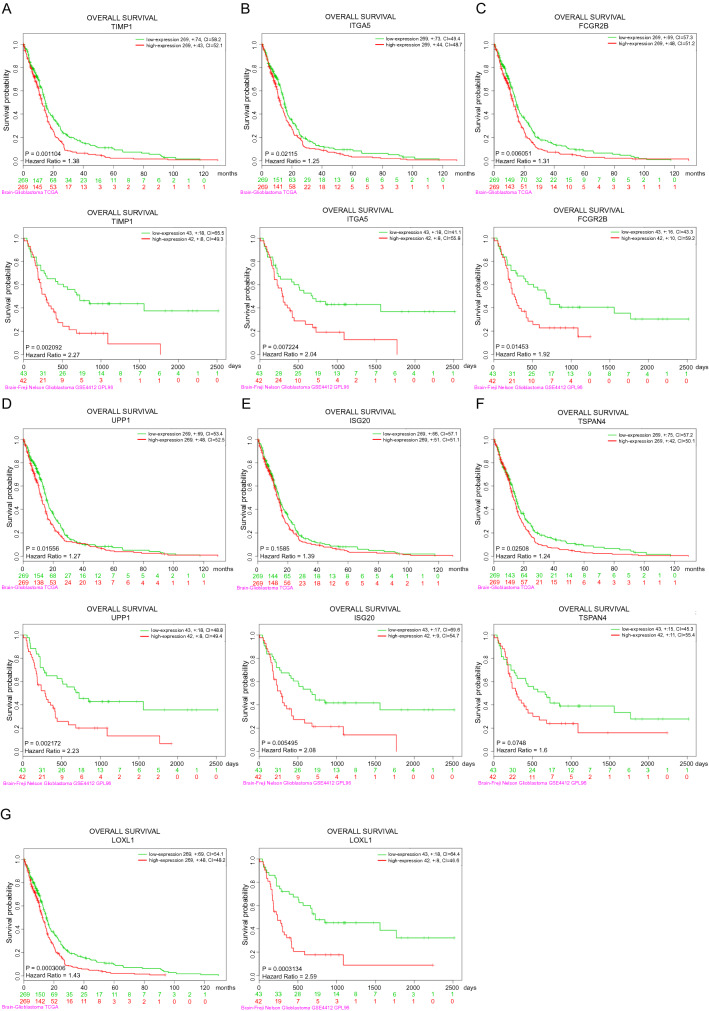
The correlations between the expression of the 7 genes and survival analysis of GBM in different datasets. Correlations between (**A**) TIMP1, (**B**) ITGA5, (**C**) FCGR2B, (**D**) UPP1, (**E**) ISG20, (**F**) TSPAN4, and (**G**) LOXL1 and the overall survival rates of GBM patients in Glioblastoma Multiforme TCGA and GSE4412. The X-axis indicates the time in months or days. Green lines indicate low expression of genes and red lines indicate high expression of genes. The groups between low and high expression of genes were stratified according to the median expression level of these genes. Data were analyzed by log-rank test.

In addition, we used the SurvExpress analysis to further assess the prognostic value of TIMP1, ITGA5, FCGR2B, UPP1, ISG20, TSPAN4, and LOXL1 using other glioblastoma patient cohorts, including TCGA Glioblastoma and GSE4412. Most of these seven genes displayed significant correlations with the survival of the glioblastoma patients in other databases examined (Fig. 4).

### Correlations of TIMP1 level with survival and immune cell infiltration in the pan-cancer analysis

Earlier, we found that seven genes are associated with both prognosis and immune cell infiltration in glioblastoma. Amongst them, TIMP1 is one of the closest genes correlated with tumor development and immune responses^18^. TIMP1 is secreted and functions as a soluble protein, but its oncogenic role is not well understood^18^. Hence, TIMP1 was selected for further characterization.

To determine whether TIMP1 is differentially expressed in cancer, we found that TIMP1 actually upregulated in the majority of tumor types in comparison to normal tissues in Oncomine database (Fig. S1A,B). We then evaluated the prognostic value of TIMP1 and used PROGgeneV2 to investigate the potential correlations between TIMP1 level and survival outcome of cancer patients. As a result, we found higher TIMP1 levels are associated with worse survival rates (Fig.S2, S3 and Table S2).

Furthermore, we assessed the association between TIMP1 and tumor-infiltrating cells among multiple forms of human cancer in TIMER database. Analysis through GENE module revealed that TIMP1 expression significantly correlates with tumor purity, the levels of B cells, CD8+ T cells, CD4+ T cells, macrophages, neutrophils, and dendritic cells (Table S3). Importantly, when cross-referencing the results shown in Figure 4, we discovered that TIMP1 serves as potential biomarker for both survival and immune infiltration in GBM, STAD, HNSC, and LGG. More specifically, TIMP1 positively correlates with the levels of dendritic cell infiltration in GBM, STAD, HNSC, and LGG and with the levels of macrophage infiltration in STAD and HNSC. Moreover, high levels of TIMP1 are associated with high neutrophil infiltration in LGG (Fig. S4).

To investigate further the relationships between TIMP1 and the diverse tumor-infiltrating immune cells, we studied the correlations between TIMP1 expression levels and those of immune markers for various immune cells in GBM, STAD, HNSC, and LGG using the CORRELATION Module in the TIMER database. We found significant positive correlations between TIMP1 expression and 44 and 47 out of the total 57 immune markers in STAD and LGG, respectively. Moreover, 18 and 41 out of the total 56 immune markers in GBM and HNSC were significantly correlated with TIMP1 expression, respectively (Table S4).

Similarly, cross-examination of STAD and LGG patient cohorts in GEPIA database, revealed significant correlations between the expression of TIMP1 and that of immune markers in CD8+ T cells, T cells, B cells, monocytes, tumor-associated macrophages, and other macrophages, indicating that TIMP1 may promote the polarization of macrophage in STAD and LGG tumors (Fig. S5 and Table 2).

**Table 2:** The correlations between TIMP1 expression and immune marker sets in GEPIA.

Description	Signature	STAD				LGG	
		Tumor		Normal		Tumor	
		R	P	R	P	R	**P**
CD8 + T cell	CD8A	0.23	3.8e −06	0.034	0.84	0.48	1.7e−31
	CD8B	0.17	0.00074	− 0.035	0.84	0.28	6.4e−11
T cell (general)	CD2	0.24	1.2e−06	− 0.065	0.71	0.6	6.2e−52
	CD3D	0.22	1.2e−05	− 0.077	0.66	0.53	5.2e−39
	CD3E	0.23	2.9e−06	− 0.013	0.94	0.6	9.1e−52
B cell	CD19	0.035	0.48	0.14	0.41	0.37	2.3e−18
	CD79A	0.15	0.0026	− 0.061	0.72	0.14	0.0014
Monocyte	CD115(CSF1R)	0.37	9.3e−15	0.4	0.015	0.055	0.21
	CD86	0.39	5.5e−16	0.31	0.07	0.25	7.6e−09
TAM	CCL2	0.51	5.1e−28	0.58	0.00026	0.38	2.3e−19
	CD68	0.25	2.4e−07	-0.12	0.48	0.28	1.5e−10
	IL10	0.35	4.7e−13	0.4	0.017	0.28	9e−11
M1 Macrophage	COX2(PTGS2)	0.21	1.2e−05	0.6	0.00014	0.27	4.2e−10
	INOS(NOS2)	-0.091	0.066	0.17	0.33	− 0.014	0.76
	IRF5	0.2	6.5e−05	− 0.28	0.1	0.24	5.4e−08
M2 Macrophage	CD163	0.47	8.2e−24	0.75	7.8e−07	0.46	1.8e−28
	MS4A4A	0.44	1.1e−20	0.74	2.2e−07	0.25	1e−08
	VSIG4	0.47	4.8e−24	0.77	4.9e−07	0.13	0.004

### TIMP1 level associates with poor prognosis in GBM and STAD patients

**Figure 5:**
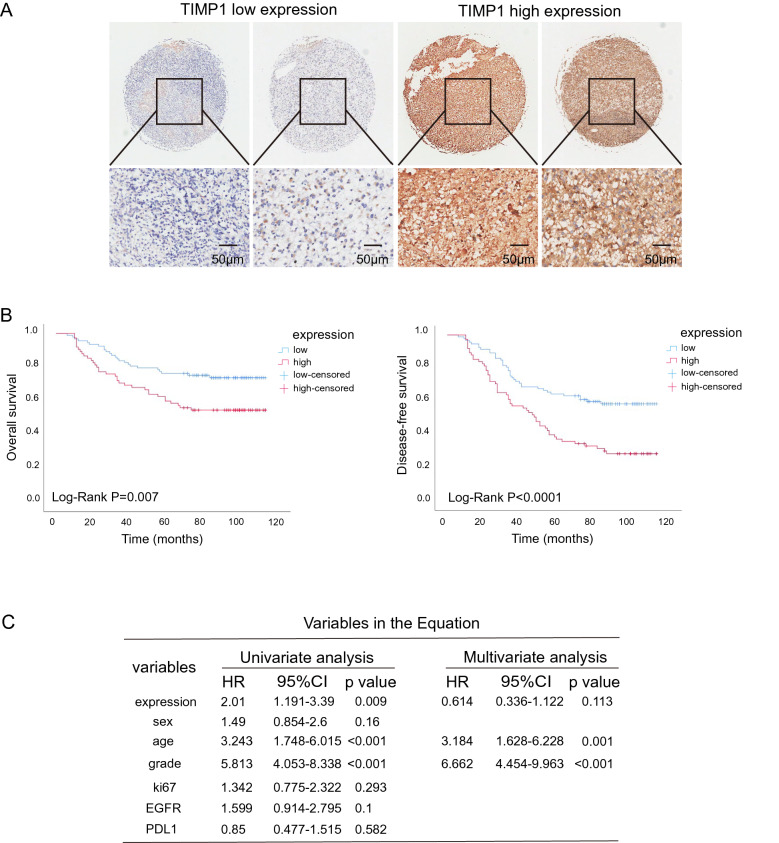
Up-regulated TIMP1 expression is associated with poor outcomes of GBM. (**A**) Representative IHC images of TIMP1 staining in GBM tumors (magnification, ×3  and ×20 ). Blue color indicates staining for nuclei and brown color indicates staining for TIMP1 protein. (**B**) Kaplan–Meier analysis of overall survival and diseasE−free survival of 180 glioma patients. The low expression and high expression of TIMP1 were grouped by the IHC total score. Data were analyzed by log-rank test. (**C**) Univariate and multivariate regression analyses of TIMP1 for overall survival in glioma patients. *Ki67* antigen identified by monoclonal antibody Ki-67, *EGFR* epidermal growth factor receptor, *PDL1* programmed cell death protein−1.

Previously, we assessed the expression of TIMP1 in GBM and STAD patients through Oncomine database (Fig. S1A,B). Moreover, we confirmed by IHC staining of GBM and STAD tissue microarrays (Fig. 5A and S6A,B). We evaluated the correlation of TIMP1 expression and clinicopathological characters among 180 glioma patients (Table 3) and 94 stomach adenocarcinoma patients (Table 4). The outcomes indicated that TIMP1 expression remarkably correlated with tumor encapsulation and recurrence. Furthermore, highly expressed TIMP1 can be recognized as an independent prognostic biomarker, as the p-value of overall survival (OS) was 0.007 and the p-value of diseasE−free survival (DFS) was less than 0.0001 in glioma patients (Fig. 5B). Notably, similar observations were detected in stomach adenocarcinoma patients (Fig. S6C). Besides, TIMP1 levels were also positively associated with the degree of lymph node metastasis (Fig.S6D), with the deeper the infiltration of cancer cells, the higher the level of expression of TIMP1 (Fig. S6E). Notably, univariable Cox regression analysis revealed that TIMP1 is significantly associated with poorer outcomes (Fig. 5C and S6F). Altogether, our results demonstrated that high TIMP1 expression is tightly linked to the worse prognosis of GBM and STAD patients.

**Table 3:** The correlation of TIMP1 expression and clinicopathological characters among 180 glioma patients.

Variables	TIMP1 expression		Total	χ2	p value
	Low	High			
**Age (year)**				5.324	0.021
≤ 40	48	25	73		
> 40	45	49	94		
Null					
**Sex**				1.201	0.273
Female	32	32	64		
Male	61	43	104		
Null					
**Grade**				9.182	0.002
HGG	66	36	102		
LGG	27	39	66		
**Grade**				12.921	0.005
1	33	15	48		
2	33	21	54		
3	21	22	43		
4	6	17	23		
**EGFR**				2.4	0.121
Negative	42	25	67		
Positive	50	49	99		
Null					
**Ki-67**				8.601	0.003
Negative	45	20	65		
Positive	47	55	102		
Null					
**PD-L1**				30.772	< 0.001
Negative	81	35	116		
Positive	12	39	51

**Table 4:** The correlation of TIMP1 expression and clinicopathological characters among 94 stomach adenocarcinoma patients.

Variables	**TIMP1** expression		Total	χ^2^	p value
	Low	High			
**Age (year)**				0.002	0.961
< 70	12	31	43		
≥ 70	14	37	51		
Null					
**T stage**				0.816	0.366
T1/T2	6	9	15		
T3/T4	19	58	77		
**TNM stage**				4.696	0.03
Ι/II	14	21	35		
III/IV	11	46	57		
Null					
**N stage**				4.546	0.033
N0	10	12	22		
N1/N2/N3	16	56	72		
Null					
**Size**				0.213	0.645
≤ 5 cm	12	35	47		
> 5 cm	14	33	47		
Null					
**Sex**				0.396	0.529
Female	11	24	35		
Male	15	44	59		
Null					
**Vessel invasion**				0.044	0.835
No	17	46	63		
Yes	9	22	31		
**Pathologic type**				3.994	0.136
AD	21	43	64		
MuA	3	7	10		
SRCC	2	18	20		
**Grade**				0.017	0.898
II	8	20	28		
III/IV	18	48	66		
**CD8**				0.006	0.937
Negative	10	27	37		
Positive	15	39	54		
**PD1**				0.247	0.619
Negative	13	30	43		
Positive	12	35	47		
**PDL1**				0.002	0.967
Negative	12	32	44		
Positive	13	34	47		
**HER2**				1.196	0.274
Negative	26	62	88		
Positive	0	6	6		
**MLH1**				1.414	0.234
Negative	15	29	44		
Positive	11	37	48		
**MSH2**				0.046	0.83
Negative	15	37	52		
Positive	11	30	41		
**MSH6**				0.36	0.549
Negative	13	28	41		
Positive	13	37	50		
**PMS2**				0.015	0.901
Negative	11	30	41		
Positive	14	36	50		

*Fisher's exact test.

### Identification of the transcription factor Sp1 in TIMP1 regulation

**Figure 6:**
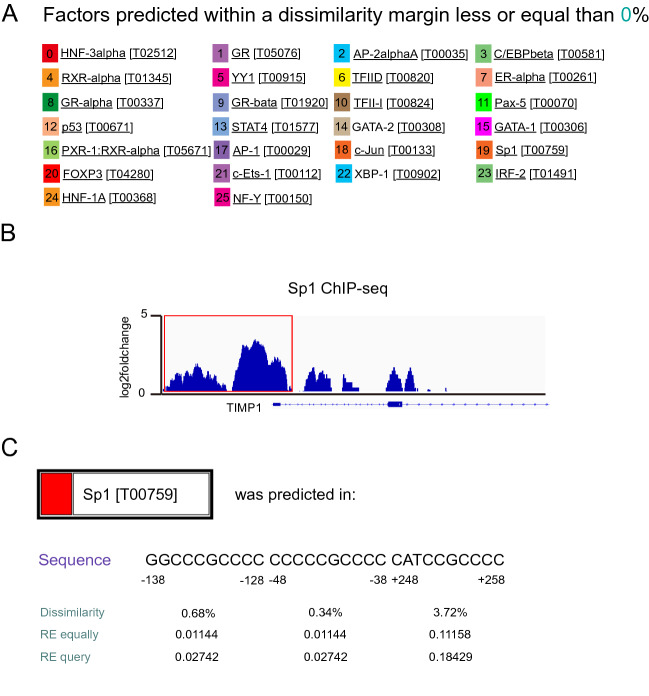
Identifying transcriptional factors of TIMP1. (**A**) Prediction of the upstream transcriptional factor of TIMP1 in PROMO database (maximum matrix dissimilarity rate—0.5). (**B**) Sp1 ChIP-seq peak of TIMP1 promoter region from GEO database (GSE92217). The signal in the red rectangle indicates Sp1 interacts with the promoter region of TIMP1. (**C**) Prediction binding sites between Sp1 and TIMP1 promoter (−2000 bp to  + 1000 bp) by PROMO database. Binding sites 1, −138 bp  to −128 bp; Binding sites 2, −48 bp to −38 bp; Binding sites 3, + 248 bp to  + 258 bp.

After demonstrating the prognosis biomarker value of TIMP1 by IHC staining of glioma patients, we aimed to investigate the upstream transcriptional factor of TIMP1 in GBM. Twenty-six transcriptional factors were predicated on regulating TIMP1 by using TIMP1 promoter DNA sequences (−2000 bp ~ + 1000 bp) in PROMO database (Fig. 6A). Next, we assessed the potential correlation of the expression levels of the transcriptional factors with TIMP1 expression level, the survival and the differential expression in GBM patients from the TCGA and CGGA databases. The results indicated Sp1 is simultaneously in line with the three conditions (Fig. S7A–D). Besides, Sp1 is over-expressed in brain glioma cancer and can facilitate proliferation and invasion of glioma cells^47^. More importantly, Sp1 correlates with immune cell infiltration, which corresponds with the results of TIMP1 of our previous findings. As a consequence, Sp1 was selected for further investigation. Sp1 ChIP-seq data from HEK293T cell (GSE92217) in GEO database was examined, and the result indicated a binding signal exists within the promoter region of *TIMP1* (Fig. 6B). We then used the PROMO database to predicate the Sp1 binding sites within the *TIMP1* promoter and identified 3 putative binding sites, BS1 (−138 to −128), BS2 (−48 to −38), BS3 (+248 to +258) (Fig. 6C).

### Sp1 binds to TIMP1 promoter and enhances TIMP1 expression

**Figure 7:**
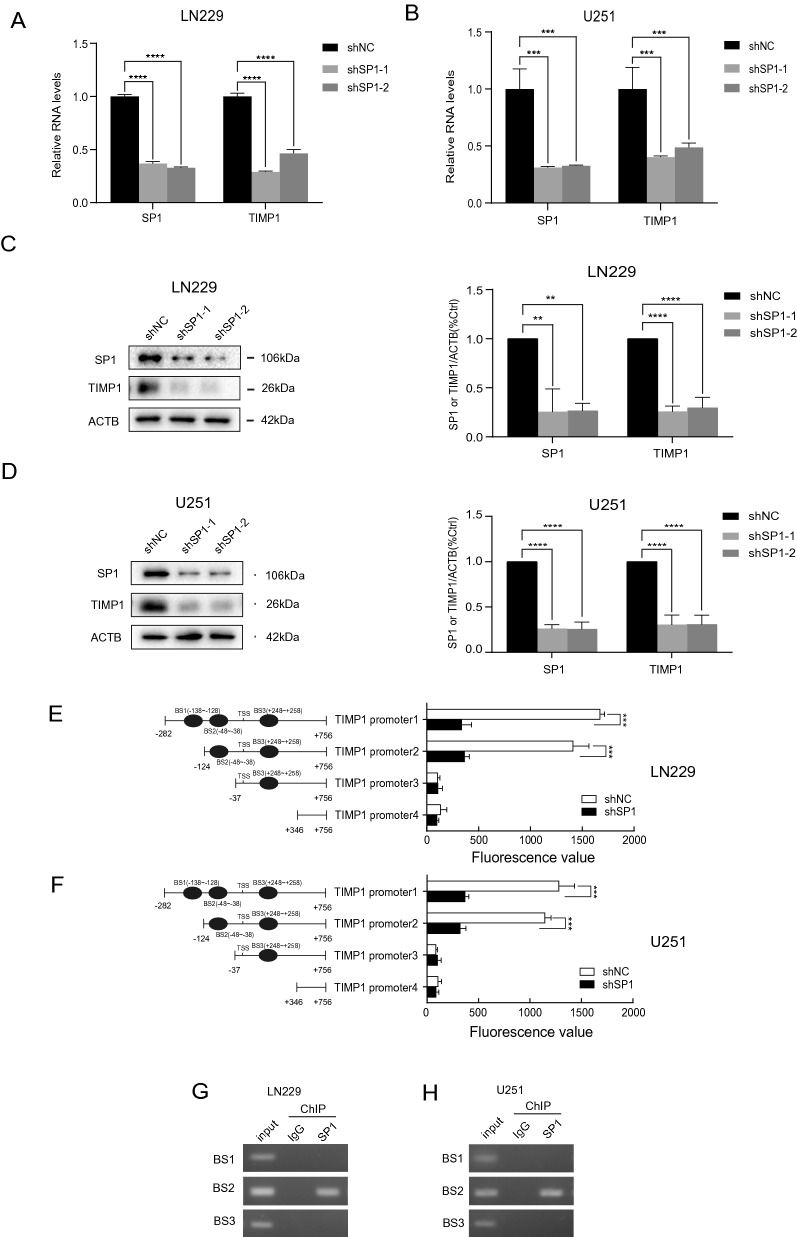
Sp1 binds to the TIMP1 promoter region and regulates TIMP1 expression in GBM. (**A**) Sp1 and TIMP1 mRNA levels in LN229 cells with stable Sp1 knockdown were detected by qPCR. n = 3 independent experiments. (**B**) Sp1 and TIMP1 mRNA levels in U251 cells with stable Sp1 knockdown were detected by qPCR. n = 3 independent experiments. (**C**) Sp1 and TIMP1 protein levels in LN229 cells with stable Sp1 knockdown were detected by western blot. ACTB was blotted as an internal (loading) control. n = 3 independent experiments. (**D**) Sp1 and TIMP1 protein levels in U251 cells with stable Sp1 knockdown were detected by western blot. ACTB was blotted as an internal (loading) control. n = 3 independent experiments. (**E,F**) Fragments of TIMP1 promoter in the luciferase reporter plasmids (left). The activity of the different fragments of TIMP1 promoter was determined by dual-luciferase assay, n = 3 independent experiments (right); (**G,H**) Binding of Sp1 to TIMP1 promoter was examined by ChIP assay. Data were presented as mean ± s.d. of three independent experiments. (**A,B**) and (**C,D**) Right panel were analyzed by onE−way ANOVA + two-side Dunnett test, **P < 0.01, ***P < 0.001, ****P < 0.0001. (**E,F**) were analyzed by two-side Student’s *t*-tset, ***P < 0.001.

To identify the potential role of Sp1 on TIMP1 expression, we constructed Sp1 shRNAs to study their effects on TIMP1 expression. Both the RNA and protein levels of TIMP1 were decreased in response to Sp1 knockdown in glioma cells (Fig. 7A–D). Next, we developed luciferase reporter plasmids, including three predictive binding sites, to detect the efficacy of TIMP1 transcription. The results indicated that the *TIMP1* promoter region responds to Sp1 at the −282/ +756bp (Fig. 7E,F). To further validate this finding and map the direct binding site, chromatin precipitation (ChIP) was performed. The result showed that Sp1 could interact predominantly with the binding site −48/ −38 of the *TIMP1* promoter (Fig. 7G,H). Altogether, our findings indicated that Sp1 binds to the promoter of *TIMP1* to enhance its expression.

### The ratio of Sp1 to TIMP1 is a better biomarker to predict the prognosis of GBM

**Figure 8:**
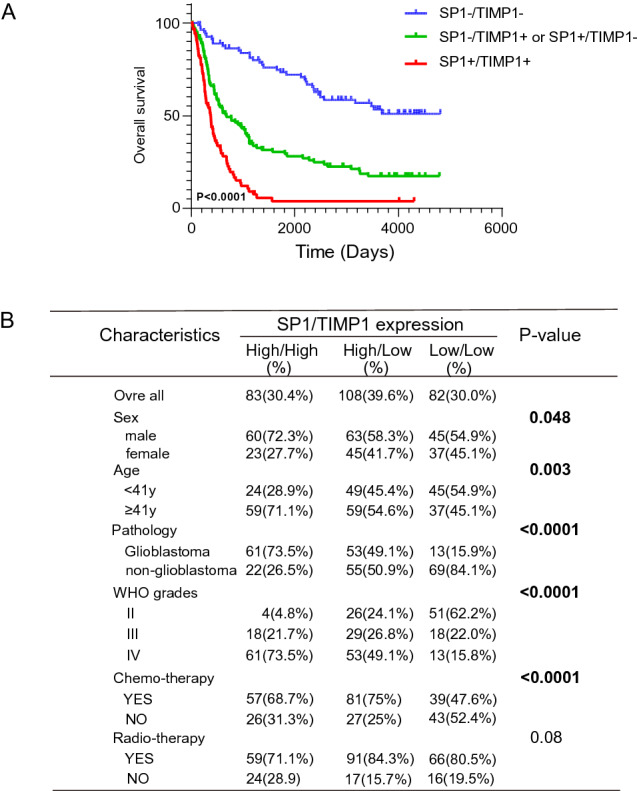
Sp1 and TIMP1 as a common biomarker to predict the prognosis of GBM. (**A**) OS of expression for both TIMP1 and Sp1 from patients in CGGA database. The blue line indicates bothe Sp1 and TIMP1 low expression, red lines indicate bothe Sp1 and TIMP1 high expression, and green lines indicate either Sp1 high expression or TIMP1 high expression. The groups were stratified according to the median expression level of Sp1 and TIMP1. Data were analyzed by log-rank test. (**B**) Relations between Sp1 and TIMP1 expression levels and clinicopathologic characteristics in CCGA database( mRNAseq 325). Significant values are in bold.

Either Sp1 or TIMP1 was highly expressed in glioblastoma patients and correlated with poor prognosis. However, whether the signature of Sp1 and TIMP1 could be a better predictive biomarker is unclear. Therefore, we evaluated the relationship between OS and the group based on TIMP1 and Sp1 expression (Sp1^high^/TIMP1^high^, Sp1^high^/TIMP1^low^, Sp1^low^/TIMP1^high^ or Sp1^low^/TIMP1^low^ groups) in GBM patients from CGGA database. The results indicated that high expression of TIMP1 and Sp1 correlate with poor prognosis (Fig. 8A). Relations between the levels of Sp1 andTIMP1 expression and clinicopathologic characteristics also confirm that the combination of Sp1 and TIMP1 could be better in predicting prognosis than the individual marker alone (Fig. 8B and S8A–G). In addition, the significant differences of overall survival between no chemo-/radiotheray and chemo-/radiotherapy indicates gliomal patients may benefit from the treatment based on the expression levels of TIMP and Sp1 (Fig S7E,F).

## Discussion

TIME plays an essential role in the development of GBM. It has been reported that TIME has both positive and negative effects on cancer development, and these function correlates with poor prognosis and therapy response in GBM patients^19^. However, there is still a lack of reliable biomarkers to predict both the prognosis and immune infiltration in glioma patients. Through analyzing patients’ clinical information and genetic profiles from online databases, TIMP1, ITGA5, FCGR2B, UPP1, ISG20, TSPAN4, and LOXL1 were identified as potential prognostic biomarkers for glioblastoma. TIMP1 is a specific in-hibitor of matrix metalloproteinase, and the aberrant upregulation of TIMP1 exists in different types of cancers^22^. TIMP1 increases proliferation and metastasis in pancreatic and colorectal cancer^26,30^. Besides, TIMP1 also has a close relationship with immunity. For example, the peripheral level of TIMP1 could stimulate the granulopoiesis in the bone marrow of mice^54^. In our research, TIMP1 has been consistently identified to be a predictive factor of both prognosis and immune infiltration in our differential gene expression analyses. Our results show that high TIMP1 levels are positively associated with increased immune infiltration levels of tumor-infiltrating lymphocytes, which could migrate towards the tumor tissues in glioma, including LGG and GBM.

Additionally, TIMP1 expression is also correlated with immune markers, especially those commonly seen in macrophages that are in M1 or M2 phase, suggesting that TIMP1 may have regulative effects on tumor-associated macrophage polarization. These polarized macrophages engage the tumor growth and progression by providing adaptive immunity and inflammatory circuits. There were several researchers also reported the prognostic value of TIMP1. TIMP1 was recognized as a serum biomarker of colorectal cancer through meta-analysis^31^. It also exerts the same role in breast cancer^32^. Moreover, a recent study investigated the correlation between the TIMP family and immune infiltration in glioblastoma; however, it only focused on the TIMP family and lacked further validation ^55^. Compared with previous studies, we evaluated the prognosis of glioma patients in large sample data and selected the genes correlating with survival. Then, these targeted genes were used to identify the relationship with tumor immune infiltration, followed by IHC validation of clinical sample chips. Therefore, our screening process is more reasonable and reliable than others. Although this relationship of TIMP1 and glioma patients was confirmed by IHC, we will further validate this result in vivo.

Transcriptional factors regulate various gene expression to mediate the malignant progression of tumors. However, the transcription factors regulating TIMP1 expression are still not uncovered in GBM. In our research, we found Sp1 could trigger the transcription of TIMP1 in GBM. Through prediction from the PROMO database, we identified 26 candidates that can potentially regulate TIMP1. Furthermore, to narrow the number down, we integrated the information of these transcriptional factors with their expression, survival analysis and correlation with TIMP1 in glioma patients. Among thesescreened transcriptional factors, Sp1 was over-expressed in most tumor cells and tissues. In addition, further studies also reported Sp1 regulates the inflammation and the immune cell infiltration of cancers. For example, Mina is an Sp1 regulated gene that functions in inflammation and immunity^56^.

In our study, we investigated the relationship between Sp1 and TIMP1 through database analysis and in vitro experiments. The results indicated Sp1 binds to the *TIMP1* promoter region and upregulates its expression in GBM. The transcriptional regulation mechanisms of TIMP1 were also investigated. TIMP1 could be decreased by OGG−1 in response to oxidative stress in human airway epithelial cells^57^. Similarly, TWIST1 could also downregulate TIMP1 mRNA levels in SCCBHY cells^58^. We discovered that immune response relative transcription factors Sp1 could regulate TIMP1 expression in GBM. Our finding suggests a strategy for targeting TIMP1, which is short of an applicable drug in glioma patients, through inhibiting Sp1. There are several small molecular inhibitors that could decrease the expression or activity of Sp1, including WP631, and Doxorubicin etc ^59,60^. While not hitherto applied in the clinic, some experimental evidence indicated that inhibiting Sp1 can be a feasible strategy in targeting GBM in human cancer cell lines and PDX models^61^. However, the function and the cooperation of Sp1 and TIMP1 in immune infiltration warrants further studies.

Both cancer cells and stroma cells can express TIMP1^22^. Currently, a number of studies have indicated TIMP1 is highly expressed in cancer cells and its high expression correlates with poor prognosis of patients. However, there are also some reports on TIMP1 expression and regulation in immune cells. For example, IL−19 has been shown to regulate TIMP1 expression through toll-like receptor 2 in macrophages^62^. Lipopolysaccharide (LPS) can also activate TIMP1 expression to inhibit macrophage’ function during HCV infection^63^. Sp1 are also be expressed in many cell types due to its general transcriptional function. In addition to cancer cells, Sp1 can transcriptionally regulate T-BET expression, a main regulator of IFN-γ, in NK cells and T cells^64^. Therefore, the function and regulation of TIMP1 and Sp1 in immune cells can have a vital role in tumor pregression and aslo warrants further investigation.

Most research on biomarkers focuses on single molecule to predict the prognosis of cancers. Hitherto, due to heterogeneity of cancers in individuals single predictive biomarkers cannot accurately predict the clinical outcome. As the tight regulatory relationship between Sp1 and TIMP1 has been identified here, we aim at investigating both molecules simultaneously as a diagnostic signature in assessing the prognosis of glioma patients. Our preliminary result indicates that TIMP1 and Sp1 together could be a better and more reliable biomarker than either alone in terms of prognostic value of patient survival and clinical characterization.

## Conclusion

We analyzed online datasets in Oncomine, TIMER, and some other database comprehensively to identify potential biomarkers for prognosis and immune infiltration in tumor patients, and found that TIMP1 associates not only with prognosis of GBM but also with the levels of immune infiltration seen in those patients. Moreover, database analyses and in vitro experiments demonstrated Sp1 binds to the *TIMP1* promoter and enhances TIMP1 expression in GBM. Therefore, the Sp1-TIMP1 axis could be a potential biomarker in clinical applications. Future studies of TIMP1 in larger patient cohorts are necessary to evaluate its value as a reliable biomarker in GBM patients. The potential impact of TIMP1 on lymphocytes, especially regulation of immune genes and other factors, also remains to be determined.

## Methods

### Oncomine database

Oncomine database is a large tumor gene chip database (https://www.oncomine.org). It was used to study TIMP1 levels between tumor tissues and normal tissues. The P-value <0.001 and 2 fold difference was defined as statistical significant difference.

### TIMER database

TIMER is an integrated assets to assess immune infiltration generally from different tumor types (https://cistrome.shinyapps.io/timer/). We explored TIMER based on deconvolution-based tool to investigate tumor-infiltrating cell changes through the gene expression profiles of data derived from TCGA. We evaluated TIMP1 levels among diverse tumors, and the relationship between TIMP1, ITGA5, FCGR2B, UPP1, ISG20, TSPAN4, and LOXL1 levels and the amount of immunE−infiltrating cells, consisting of CD4+ T cells, CD8+ T cells, B cells, neutrophils, macrophages, and dendritic cells, through different gene modules. We also investigated the correlations of TIMP1 level with gene markers of tumor-infiltrating immune cells.

### PROGgeneV2 database

PROGgeneV2 (http://genomics.jefferson.edu/proggene/index.php), consisting of data from 134 cohorts from 21 cancer types, was used to analyze survival models for available covariates and study prognostic implications of different gene signatures in various cancer types.

### Establishment of risk score model

To identify the prognosis value of differentially expressed genes, least absolute shrinkage and selection operator (LASSO) regression was used to establish the risk score model to predict the survival status of glioma patients. The R package glmnet was used. After we got the differentially expressed genes, the correlation between gene expression levels and the overall survival time of patients was investigated through univariate Cox regression analysis. Thirty-three genes were obtained according to the *P*-value and HR. Notably, these genes were used to establish the risk score model and receiver operating characteristic (ROC) was used to evaluate the predictive value of the risk score model. Finally, the risk score model, included six genes, SPDYE21, MTHFS, TNFSF14, LBH, ITGB7 and TIMP1.

### Gene correlation analysis with GEPIA tool

GEPIA is an online web to analysis the data obtained from TCGA database (http://gepia.cancer-pku.cn/index.html). We used this tool to detect the correlation of genes in TIMER. GEPIA was also used to obtain survival curves, through gene expression with the log-rank test and the Mantel-Cox test in different types of cancers. Correlation analysis between gene expression profiles from given sets of TCGA cancer types was performed and the coefficient was generated through Spearman analysis.

### Immunohistochemistry

Tissue microassays were applied to explore the connections of TIMP1 with prognosis of GBM and STAD patients. These microassays were purchased from Shanghai Outdo Biotech (Shanghai, China), including 180 GBM tissues, 94 STAD, and 84 STAD corresponding adjacent stomach tissues. Informed consent was obtained from the ethics committee of Shanghai Outdo Biotech Company. All of the methods in this study were in accordance with the approved guidelines, and the experimental protocols were approved by the ethics committee of Shanghai Outdo Biotech Company. IHC was conducted using Human Brain/Gastric Cancer Tissue Chip (HBraG180Su02 and HStmA180Su19). IHC total score was generated according to this equation: IHC total score = staining intensity x staining positive rate. The staining intensity was divided into 4 grades, 0, 1, 2, 3. The staining positive rate was also divided into 4 grades equally. Finally, total score was separated into 2 groups, high expression and low expression based on the detailed data. This judgment process should be conducted by two proficient pathologists independently.

### Cell culture

The human glioblastoma cell lines LN229, U251 and the human embryo kidney cell line HEK293T of our study were obtained from American Type Culture Collection (ATCC, Manassas, VA, USA). LN229 and HEK293T were cultured in DMEM (Gibico, Thermo, Inc) consisting of 5% fetal bovine serum (Gibico, Thermo, Inc) and 1% penicillin and streptomycin (Gibico, Thermo, Inc). U251 was grown in DMEM (high glucose) consisting of 10% fetal bovine serum and 1% penicillin and streptomycin. All of these cells were grown at 5% CO_2_ and 37 ℃.

### Construction of lentiviral infected cell lines

shRNAs of Sp1 were established using PLKO.1-puro plasmid. The sequences of shRNAs were listed below: shSp1−1 5’-GCTGGTGGTGATGGAATACAT-3’, shSp1-2 5’-ATGTATTCCATCACCACCAGC-3’. HEK293T cells were transfected with shRNA vector, psPAX2 and Pmd2.G (2:1:1) to generate lentiviral particles. Then the glioma cells were cultured at an appropriate density for the addition of lentiviral and polybrene (Sigma-Aldrich, 8μg/ml). After 24 hours, puromycin were used to select positive cells.

### Western blots

Cells were collected and proteins were obtained using RIPA. BCA kit (Thermo Scientific, MA, USA) was used to ensure the concentrations. Total protein were separated with SDS-PAGE and transferred to NC membranes (Merck millipore, USA) later. Then membranes were blocked in 5% milk without fat in TBST and incubated with primary antibodies at 4 ℃ for 16 h. After being washed with TBST for three times, the blots were then incubated with secondary antibody for 2 h at room temperature. The blots were developed using ECL reagent. The following antibodies were used: Sp1 (sc420; Santa cruz; 1:1000), TIMP1 (ab211926; Abcam; 1:1000), ACTB (AC004; Abclonal; 1:3000), anti-mouse HRP (31430; Thermo; 1:3000), anti-rabbit HRP (31460; Thermo; 1:3000).

### Dual luciferase reporter assay

PGL4.15 plasmid was used to construct different promoter segments consisting of TIMP1 predictive binding sites. This assay was conducted using Dual-Luciferase Reporter Assay System Kit (E1910; Promega, Madison, WI, USA). The constructed plasmids were transfected into glioma cells. Forcontrolling transfection efficiencies, pRL-CMV (Renilla luciferase) was co-transfected. After 48 hours, the luciferase activity was analyzed using a luminometer (Promega).

### qPCR assay

Using RNAiso plus Regent (9109, Takara Bio, Japan), total RNA was extracted, and cDNA was synthesized using HiScript III RT SuperMix for qPCR kit (R323-01; Vazyme, Nanjing, China) based on the instruction. The cDNA were used as the template and diluted according to instruction (2× RealStar Green Fast Mixture (A304, Genstar, Beijing , China). The machine was MX3005P (Agilent Technologies, USA). The primers used were listed below: Sp1 forward, 5’-CCACCATGAGCGACCAAGAT-3’, Sp1 reverse, 5’-AAGGCACCACCACCATTACC-3’; TIMP1 forward, 5’-AGAGTGTCTGCGGATACTTCC-3’, TIMP1 reverse, 5’-CCAACAGTGTAGGTCTTGGTG-3’; ACTB forward, 5’-ATGTGGCCGAGGACTTTGATT-3’, ACTB reverse, 5’-AGTGGGGTGGCTTTTAGGATG-3’.

### ChIP assay

The ChIP-IT^®^ Express Chromatin Immunoprecipitation Kits (53009; Active Motif, Carlsbad, CA, USA) were used to carry out ChIP assay according to the instruction. The number of cells used was about 1x10^7^. 1% formaldehyde was used to cross-linked DNA and protein for 10 minutes at room temperature. Glycine was used for fixation reaction. After homogenizing the cells, the solution was sonicated to obtain DNA fragments. Subsequently, the solution was centrifugated and the supernatant was used for immunoprecipitation by Sp1 antibody. Finally, the eluted DNAs were used to detect the levels of interaction between Sp1 and TIMP1 promoter DNA sequences.The primer sequences were listed below: TIMP1 BS1 (−138 to −128bp), forward 5’-AGGCGGCTTTTGGAAGGAATAG-3’, reverse 5’-CCCACCATCAGTGCAGAAGC-3’; TIMP1 BS2 (−48 to −38bp), forward 5’-AGTAATGCATCCAGGAAGCC-3’, reverse 5’-GGGCCCTGCTTACCTCTGGT-3’; TIMP1 BS3 (+248 to +258bp), forward 5’-AGGCTGGAACTGCTTTCCCA-3’, reverse 5’-GAAGGAATTTGCGGGGGGAT-3’.

### Statistical analysis

The outcomes derived from Oncomine were described as P-values, fold changes, and ranks. The HR and P or Cox P values generated from log-rank test methods were used to describe the analysis of Kaplan-Meier plots, PROGgeneV2, and TIMER. The analysis of correlations among different factors was evaluated through the Spearman’s statistical methods. The strength of analysis was defined as below: 0.00–0.33 “weak”, 0.33–0.67 “moderate”, 0.67–1.0“strong”. The assay results were analysised through Graphad Prism 8. The outcomes were described as mean ± S.D. Two-sided Student’s *t*-test was used for comparison of two groups. OnE−way ANOVA plus two-sided Dunnett test was performed for analyzing multigroups (each group compared with a control group). The P-value <0.05 was defined as statistically significant.

### Ethical approval

The studies involving human participants were reviewed and approved by Ethics committee of Shanghai Outdo Biotech Company, as described in the Methods in more detail. The patients/participants provided their written informed consent to participate in this study. All methods were performed in accordance with relevant guidelines and regulations.

## Supplementary Information

Supplementary Figure S1.

Supplementary Figure S2.

Supplementary Figure S3.

Supplementary Figure S4.

Supplementary Figure S5.

Supplementary Figure S6.

Supplementary Figure S7.

Supplementary Figure S8.

Supplementary Table S1.

Supplementary Table S2.

Supplementary Table S3.

Supplementary Table S4.

Supplementary Information.

Supplementary Legends.

## Data Availability

Materials and data from the study were available.

## Abbreviations

Sp1: Specificity protein 1
TIMP: Tissue inhibitor of matrix metalloproteinase
GBM: Glioblastoma
DEG: Differentially expressed gene
TIME: Tumor immune microenvironment
BBB: Blood–brain barrier
ADAM17: A disintegrin and metalloproteinase−17
MDK: Midkine
TMZ: Temozolomide
ITGA5: Integrin subunit alpha 5
FCGR2B: *Homo sapiens* Fc fragment of IgG receptor IIb
UPP1: Uridine phosphorylase 1
ISG20: Interferon-stimulated gene 20
TSPAN4: Human tetraspanin 4
LOXL1: Lysyl oxidase−like 1
STAD: Stomach adenocarcinoma
HNSC: Head and neck squamous cell carcinoma
LGG: Lower-grade glioma
OS: Overall survival
DFS: Disease−free survival
YY1: Yin Yang 1
JUN: Jun proto-oncogene
ETS1: E26 transformation specific−1
XBP1: X-box binding protein−1
IRF2: Interferon regulatory factor 2
OGG−1: 8-Oxoguanine DNA glycosylase−1
TWIST1: Twist family BHLH transcription factor 1

## References

[1] Louis DN (2016). The 2016 World Health Organization classification of tumors of the central nervous system: A summary. Acta Neuropathol.

[2] Suzuki H (2015). Mutational landscape and clonal architecture in grade II and III gliomas. Nat. Genet..

[3] Ostrom QT (2016). CBTRUS statistical report: Primary brain and other central nervous system tumors diagnosed in the United States in 2009–2013. Neuro Oncol..

[4] Aoki K (2018). Prognostic relevance of genetic alterations in diffuse lower-grade gliomas. Neuro Oncol..

[5] Cho SY, Kim S, Kim G, Singh P, Kim DW (2019). Integrative analysis of KIF4A, 9, 18A, and 23 and their clinical significance in low-grade glioma and glioblastoma. Sci. Rep..

[6] Hsu JB, Chang TH, Lee GA, Lee TY, Chen CY (2019). Identification of potential biomarkers related to glioma survival by gene expression profile analysis. BMC Med. Genomics.

[7] Alshabi AM, Vastrad B, Shaikh IA, Vastrad C (2019). Identification of crucial candidate genes and pathways in glioblastoma multiform by bioinformatics analysis. Biomolecules.

[8] Yin W (2019). Expression profile analysis identifies a novel fivE−gene signature to improve prognosis prediction of glioblastoma. Front. Genet..

[9] Ceccarelli M (2016). Molecular profiling reveals biologically discrete subsets and pathways of progression in diffuse glioma. Cell.

[10] Tanguturi SK (2017). Leveraging molecular datasets for biomarker-based clinical trial design in glioblastoma. Neuro Oncol..

[11] Santarpia M, Karachaliou N (2015). Tumor immune microenvironment characterization and response to anti-PD−1 therapy. Cancer Biol. Med..

[12] Butt AQ, Mills KH (2014). Immunosuppressive networks and checkpoints controlling antitumor immunity and their blockade in the development of cancer immunotherapeutics and vaccines. Oncogene.

[13] Spranger S (2014). Mechanism of tumor rejection with doublets of CTLA-4, PD−1/PD-L1, or IDO blockade involves restored IL-2 production and proliferation of CD8(+) T cells directly within the tumor microenvironment. J. Immunother. Cancer.

[14] Conroy H, Galvin KC, Higgins SC, Mills KH (2012). Gene silencing of TGF-beta1 enhances antitumor immunity induced with a dendritic cell vaccine by reducing tumor-associated regulatory T cells. Cancer Immunol. Immunother..

[15] Zhang J (2019). The combination of neoantigen quality and T lymphocyte infiltrates identifies glioblastomas with the longest survival. Commun. Biol..

[16] Jarnicki AG, Lysaght J, Todryk S, Mills KH (2006). Suppression of antitumor immunity by IL-10 and TGF-beta-producing T cells infiltrating the growing tumor: influence of tumor environment on the induction of CD4+ and CD8+ regulatory T cells. J. Immunol..

[17] Engelhardt B, Vajkoczy P, Weller RO (2017). The movers and shapers in immune privilege of the CNS. Nat. Immunol..

[18] Weiss N, Miller F, Cazaubon S, Couraud PO (2009). The blood-brain barrier in brain homeostasis and neurological diseases. Biochim. Biophys. Acta.

[19] Quail DF, Joyce JA (2017). The microenvironmental landscape of brain tumors. Cancer Cell.

[20] Chen W (2015). Glioma cells escaped from cytotoxicity of temozolomide and vincristine by communicating with human astrocytes. Med. Oncol..

[21] Brew K, Dinakarpandian D, Nagase H (2000). Tissue inhibitors of metalloproteinases: Evolution, structure and function. Biochim. Biophys. Acta.

[22] Jackson HW, Defamie V, Waterhouse P, Khokha R (2017). TIMPs: Versatile extracellular regulators in cancer. Nat. Rev. Cancer.

[23] Wang YY, Li L, Zhao ZS, Wang HJ (2013). Clinical utility of measuring expression levels of KAP1, TIMP1 and STC2 in peripheral blood of patients with gastric cancer. World J. Surg. Oncol..

[24] Zurac S (2016). Variations in the expression of TIMP1, TIMP2 and TIMP3 in cutaneous melanoma with regression and their possible function as prognostic predictors. Oncol. Lett..

[25] Davidsen ML (2006). TIMP-1 gene deficiency increases tumour cell sensitivity to chemotherapy-induced apoptosis. Br. J. Cancer.

[26] D'Costa Z (2017). GemcitabinE-induced TIMP1 attenuates therapy response and promotes tumor growth and liver metastasis in pancreatic cancer. Cancer Res..

[27] Fu ZY, Lv JH, Ma CY, Yang DP, Wang T (2011). Tissue inhibitor of metalloproteinasE-1 decreased chemosensitivity of MDA-435 breast cancer cells to chemotherapeutic drugs through the PI3K/AKT/NF-small ka, CyrillicB pathway. Biomed. Pharmacother..

[28] Rhee JS, Diaz R, Korets L, Hodgson JG, Coussens LM (2004). TIMP-1 alters susceptibility to carcinogenesis. Cancer Res..

[29] Lee SY (2014). TIMP-1 modulates chemotaxis of human neural stem cells through CD63 and integrin signalling. Biochem. J..

[30] Seubert B (2015). Tissue inhibitor of metalloproteinases (TIMP)-1 creates a premetastatic niche in the liver through SDF-1/CXCR4-dependent neutrophil recruitment in mice. Hepatology.

[31] Meng C (2018). TIMP-1 is a novel serum biomarker for the diagnosis of colorectal cancer: A meta-analysis. PLoS ONE.

[32] Schmitt M, Sweep FC (2009). Tissue inhibitor metalloproteinase typE-1 (TIMP-1), a novel cancer biomarker predicting response of adjuvant anthracyclinE-based chemotherapy in patients afflicted with primary breast cancer. Eur. J. Cancer.

[33] Slater EP (2013). LCN2 and TIMP1 as potential serum markers for the early detection of familial pancreatic cancer. Transl. Oncol..

[34] Song G (2016). TIMP1 is a prognostic marker for the progression and metastasis of colon cancer through FAK-PI3K/AKT and MAPK pathway. J. Exp. Clin. Cancer Res..

[35] Wurtz SO, Schrohl AS, Mouridsen H, Brunner N (2008). TIMP-1 as a tumor marker in breast cancer–An update. Acta Oncol..

[36] Bockelman C (2018). Serum MMP-8 and TIMP-1 predict prognosis in colorectal cancer. BMC Cancer.

[37] Hawthorn L (2004). TIMP1 and SERPIN-A overexpression and TFF3 and CRABP1 underexpression as biomarkers for papillary thyroid carcinoma. Head Neck.

[38] Letovsky J, Dynan WS (1989). Measurement of the binding of transcription factor Sp1 to a single GC box recognition sequence. Nucleic Acids Res..

[39] Suske G (1999). The Sp-family of transcription factors. Gene.

[40] Wierstra I (2008). Sp1: Emerging roles–beyond constitutive activation of TATA-less housekeeping genes. Biochem. Biophys. Res. Commun..

[41] Li L, Davie JR (2010). The role of Sp1 and Sp3 in normal and cancer cell biology. Ann. Anat..

[42] Zhao C, Meng A (2005). Sp1-like transcription factors are regulators of embryonic development in vertebrates. Dev. Growth Differ..

[43] Chiefari E (2002). Increased expression of AP2 and Sp1 transcription factors in human thyroid tumors: A role in NIS expression regulation?. BMC Cancer.

[44] Wang L (2003). Transcription factor Sp1 expression is a significant predictor of survival in human gastric cancer. Clin. Cancer Res..

[45] Jiang NY (2008). Sp1, a new biomarker that identifies a subset of aggressive pancreatic ductal adenocarcinoma. Cancer Epidemiol. Biomark. Prevent..

[46] Guan H (2012). Sp1 is upregulated in human glioma, promotes MMP-2-mediated cell invasion and predicts poor clinical outcome. Int. J. Cancer.

[47] Hsu TI (2012). Sp1 expression regulates lung tumor progression. Oncogene.

[48] Luo J (2015). Transcriptional factor specificity protein 1 (SP1) promotes the proliferation of glioma cells by up-regulating midkine (MDK). Mol. Biol. Cell.

[49] Szalad A, Katakowski M, Zheng X, Jiang F, Chopp M (2009). Transcription factor Sp1 induces ADAM17 and contributes to tumor cell invasiveness under hypoxia. J. Exp. Clin. Cancer Res..

[50] Wang Z, Li Z, Fu Y, Han L, Tian Y (2019). MiRNA-130a-3p inhibits cell proliferation, migration, and TMZ resistance in glioblastoma by targeting Sp1. Am. J. Transl. Res..

[51] Beishline K, Azizkhan-Clifford J (2015). Sp1 and the 'hallmarks of cancer'. FEBS J..

[52] Zhang Y (2021). Comprehensive transcriptomic characterization reveals core genes and module associated with immunological changes via 1619 samples of brain glioma. Cell Death Dis..

[53] Xie Q (2021). Identification of a prognostic immunE-related signature for small cell lung cancer. Cancer Med..

[54] Kobuch J (2015). TIMP-1 signaling via CD63 triggers granulopoiesis and neutrophilia in mice. Haematologica.

[55] Han J, Jing Y, Han F, Sun P (2021). Comprehensive analysis of expression, prognosis and immune infiltration for TIMPs in glioblastoma. BMC Neurol..

[56] Lian S (2013). Transcriptional activation of Mina by Sp1/3 factors. PLoS ONE.

[57] Pan L (2019). Epigenetic regulation of TIMP1 expression by 8-oxoguanine DNA glycosylasE-1 binding to DNA:RNA hybrid. FASEB J..

[58] Okamura H, Yoshida K, Haneji T (2009). Negative regulation of TIMP1 is mediated by transcription factor TWIST1. Int. J. Oncol..

[59] Mansilla S, Priebe W, Portugal J (2004). Sp1-targeted inhibition of gene transcription by WP631 in transfected lymphocytes. Biochemistry.

[60] Mansilla S, Portugal J (2008). Sp1 transcription factor as a target for anthracyclines: effects on gene transcription. Biochimie.

[61] Vizcaino C, Mansilla S, Portugal J (2015). Sp1 transcription factor: A long-standing target in cancer chemotherapy. Pharmacol. Ther..

[62] Zhang H, Song B, He S (2018). Interleukin 29 activates expression of tissue inhibitor of metalloproteinase 1 in macrophages via tolllike receptor 2. Mol. Med. Rep..

[63] Fan C (2020). LPS stimulation during HCV infection induces MMP/TIMP1 imbalance in macrophages. J. Med. Microbiol..

[64] Yu J (2007). Transcriptional control of human T-BET expression: The role of Sp1. Eur. J. Immunol..

