# Supplementary figures and images for: Sp1 induced gene TIMP1 is related to immune cell infiltration in glioblastoma
Source: Sci Rep. 2022 Jul 1;12:11181. doi: 10.1038/s41598-022-14751-4 (PMC9249770; doi:10.1038/s41598-022-14751-4)

A

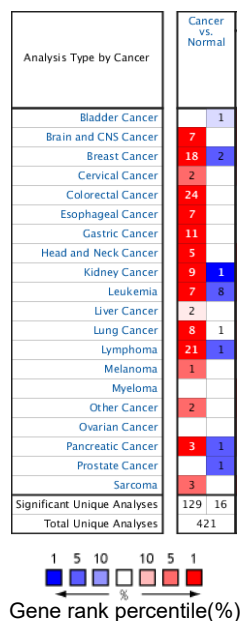

B

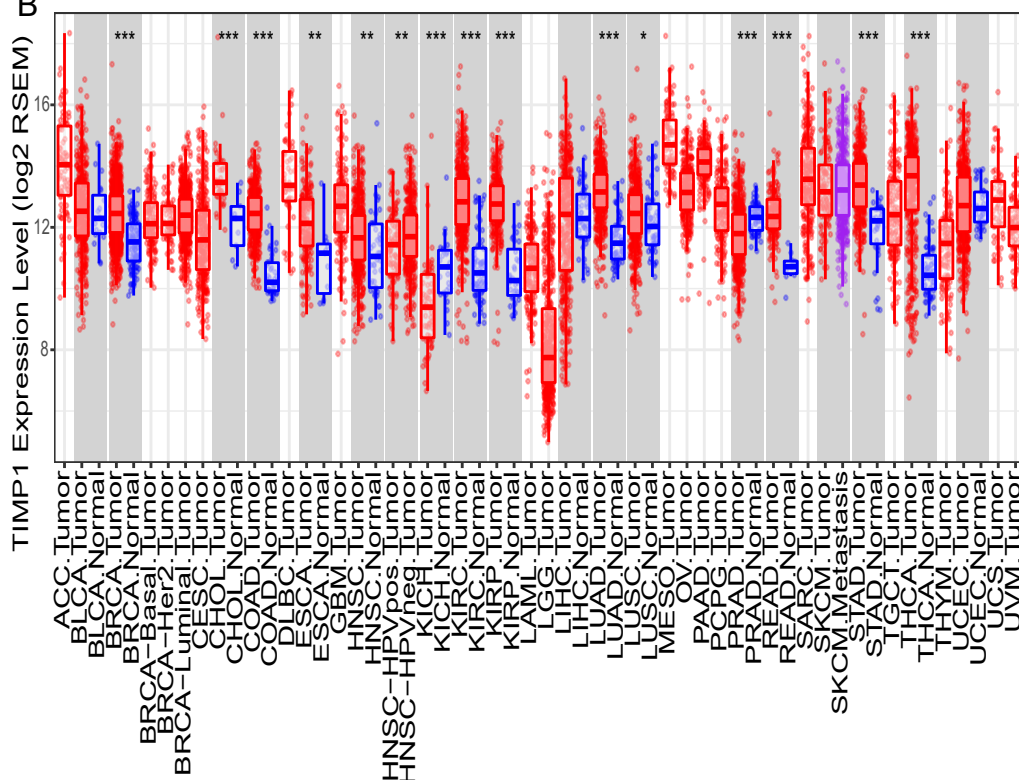

C

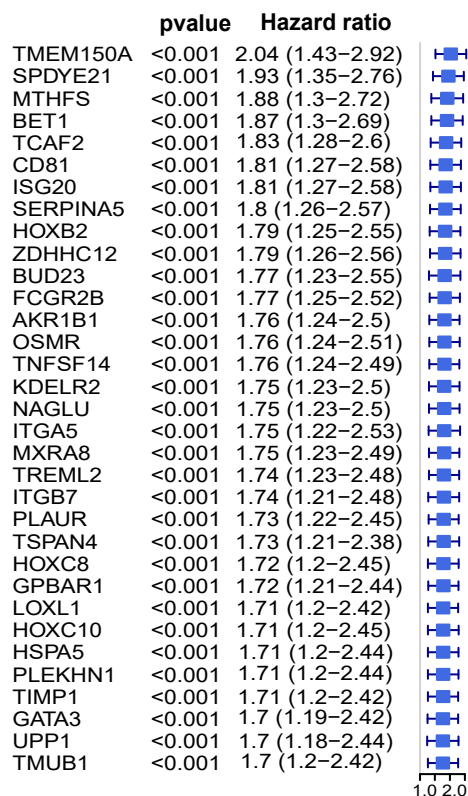

D

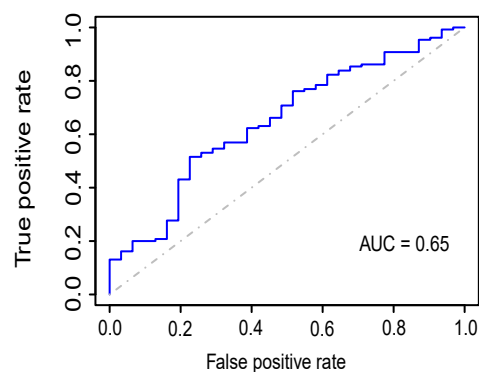

E

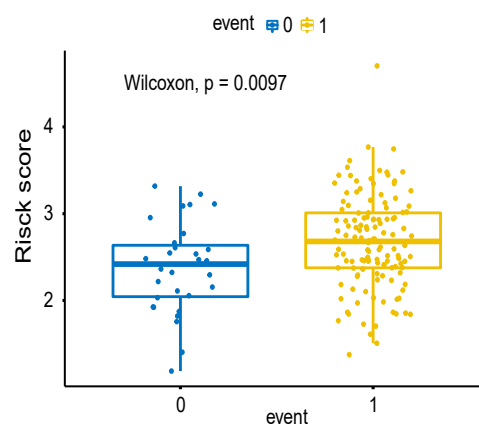

Supplement: Supplementary file 1 — Supplementary Figure S1. [file 41598_2022_14751_MOESM1_ESM.pdf]

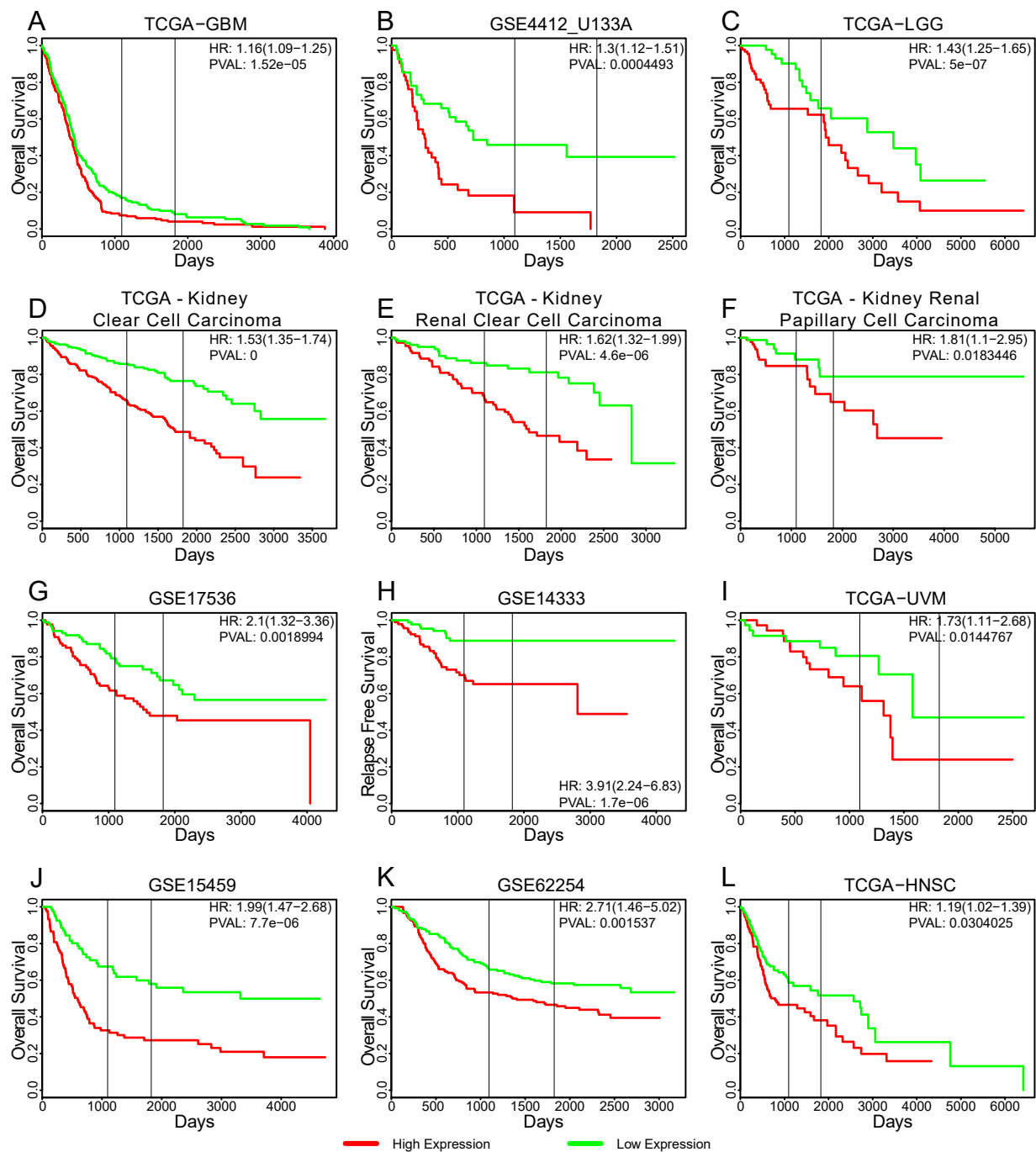

Supplement: Supplementary file 2 — Supplementary Figure S2. [file 41598_2022_14751_MOESM2_ESM.pdf]

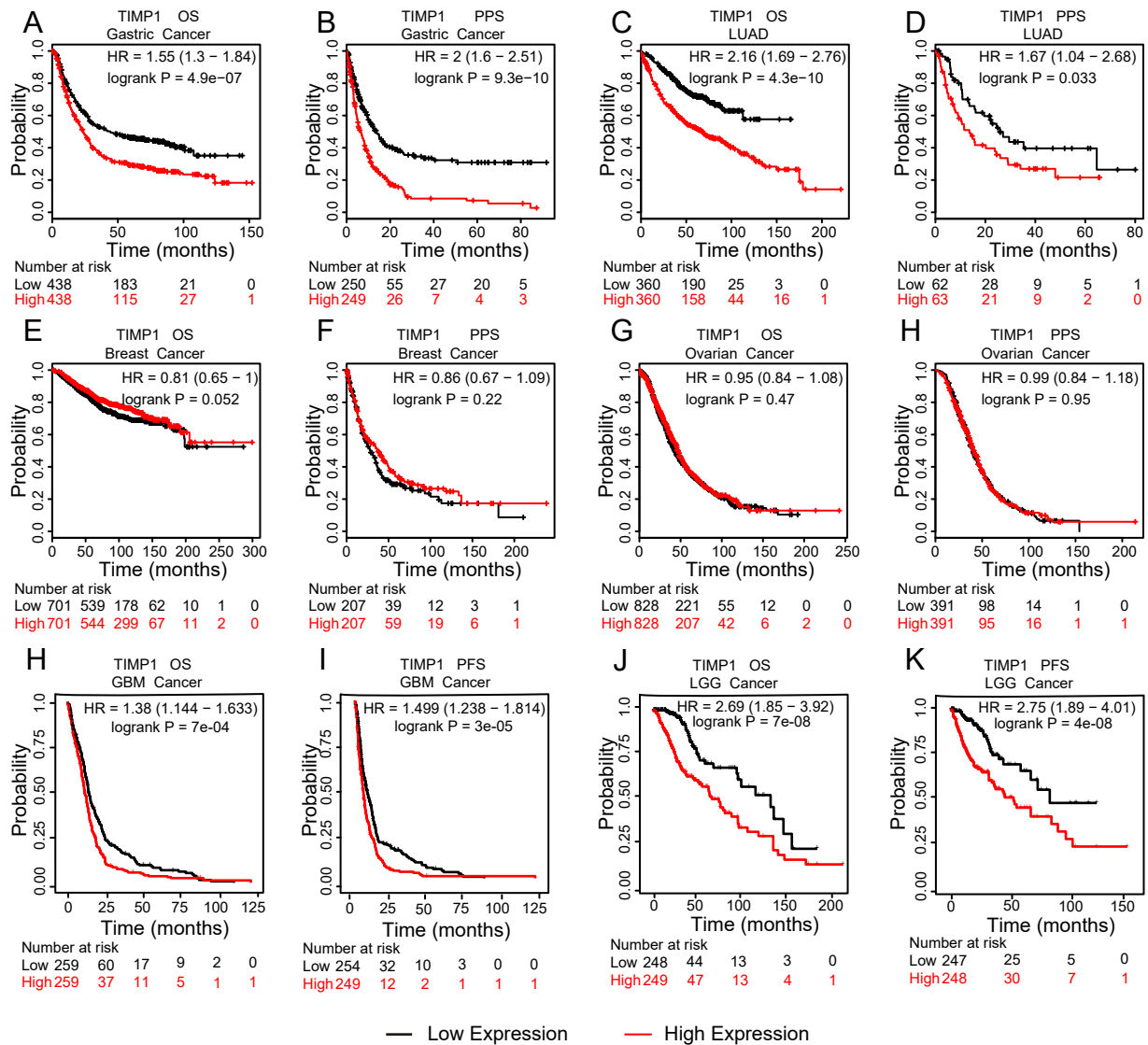

Supplement: Supplementary file 3 — Supplementary Figure S3. [file 41598_2022_14751_MOESM3_ESM.pdf]

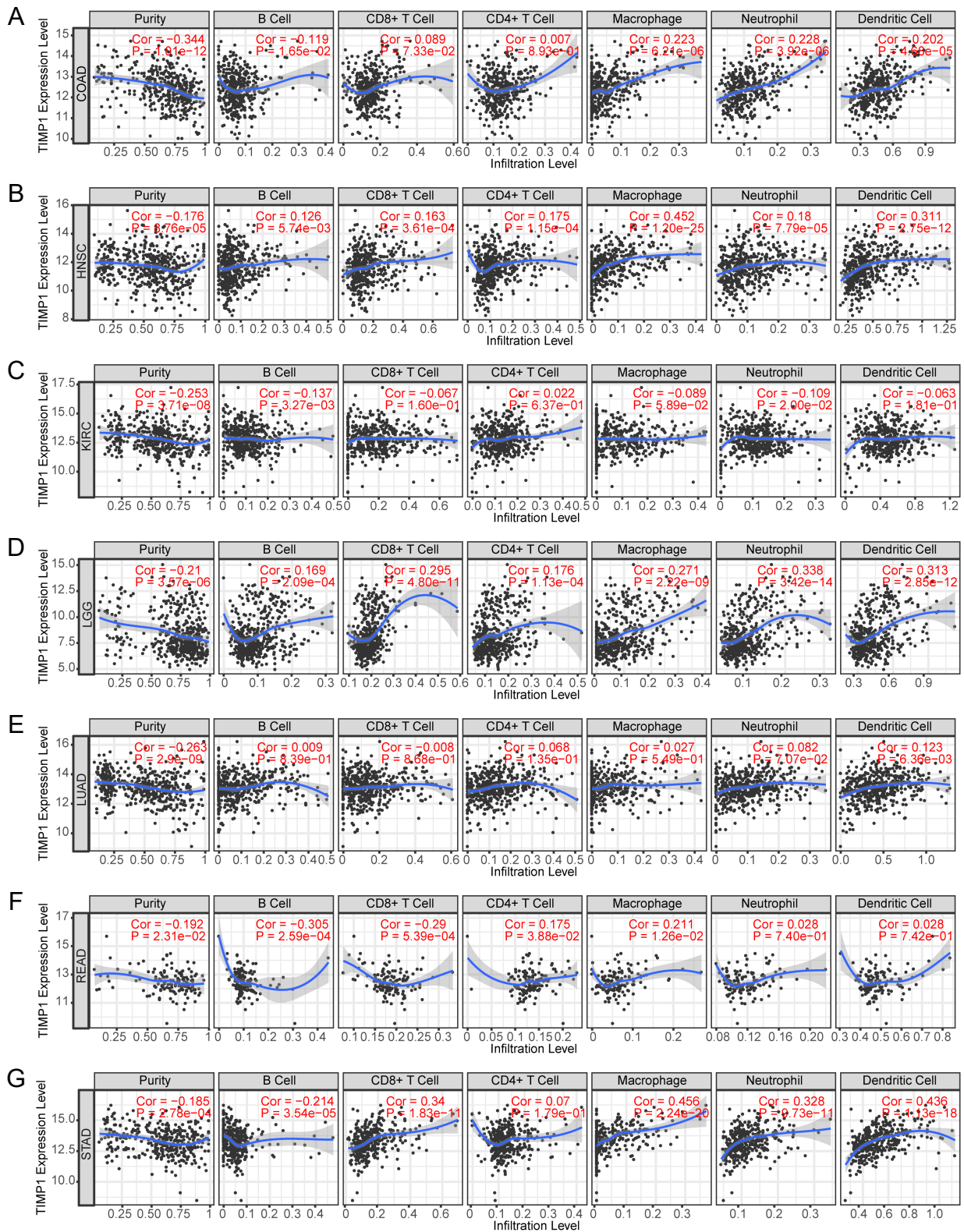

Supplement: Supplementary file 4 — Supplementary Figure S4. [file 41598_2022_14751_MOESM4_ESM.pdf]

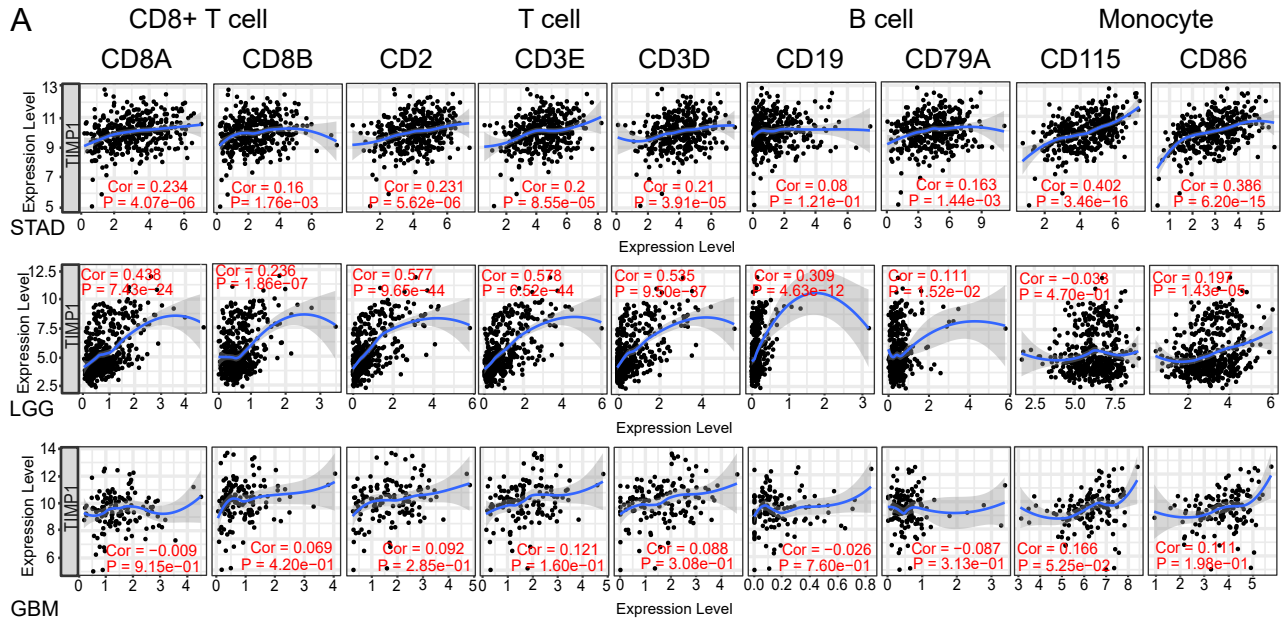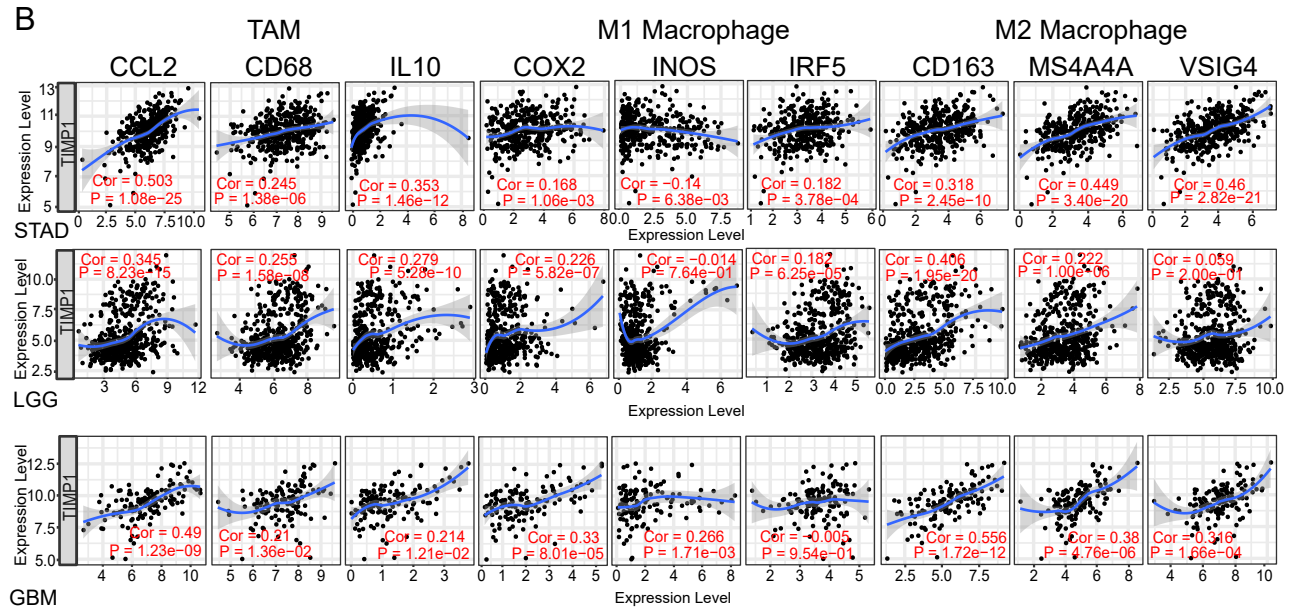

Supplement: Supplementary file 5 — Supplementary Figure S5. [file 41598_2022_14751_MOESM5_ESM.pdf]

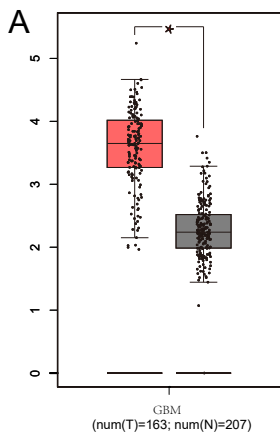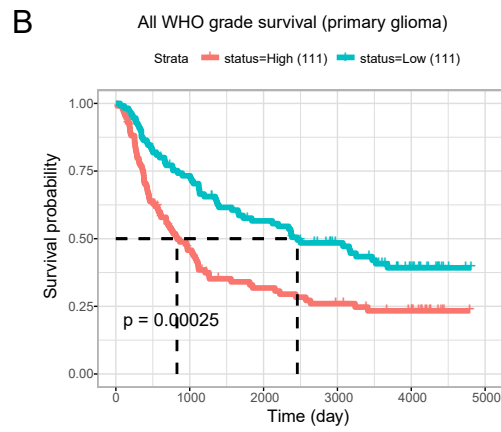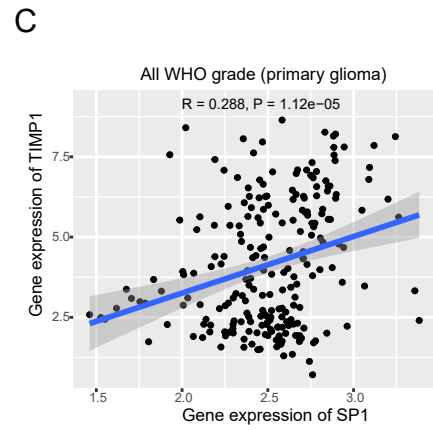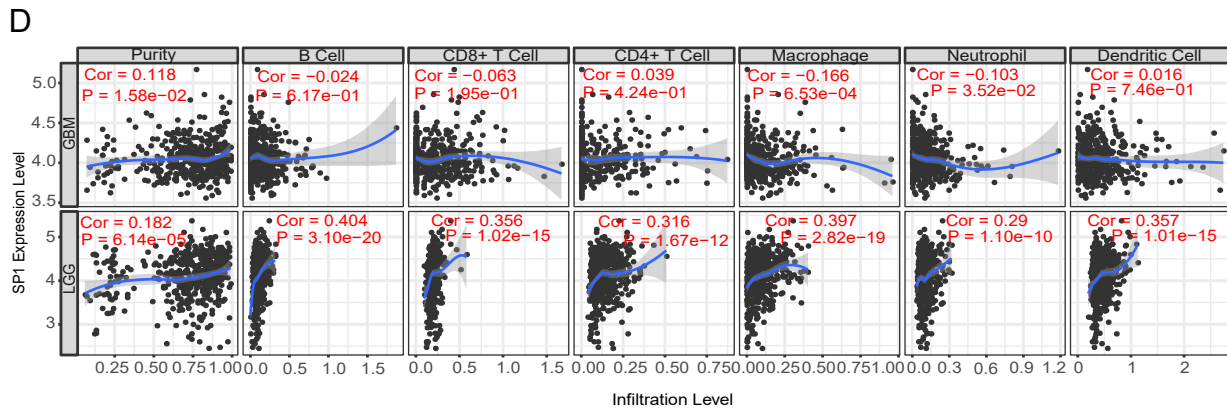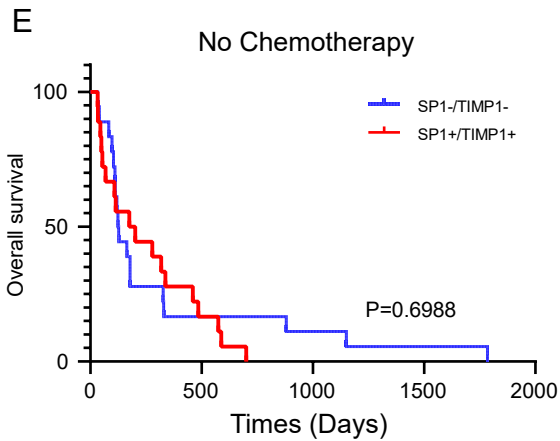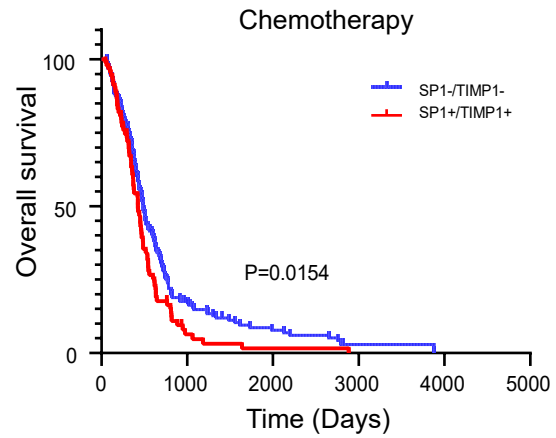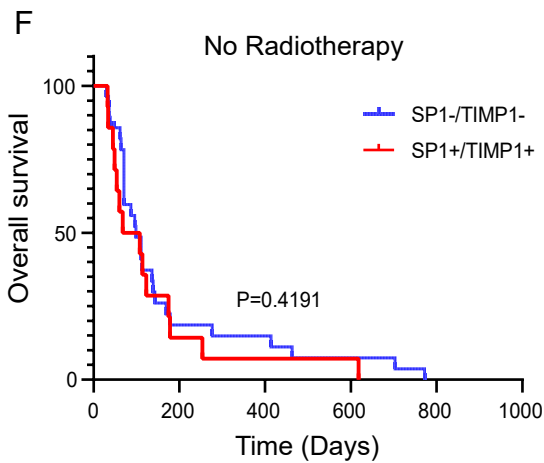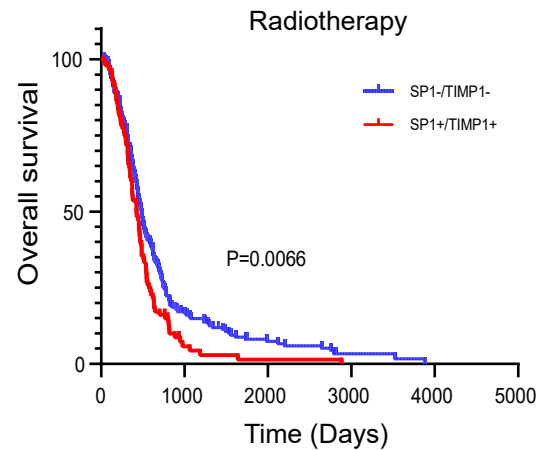

Supplement: Supplementary file 7 — Supplementary Figure S7. [file 41598_2022_14751_MOESM7_ESM.pdf]

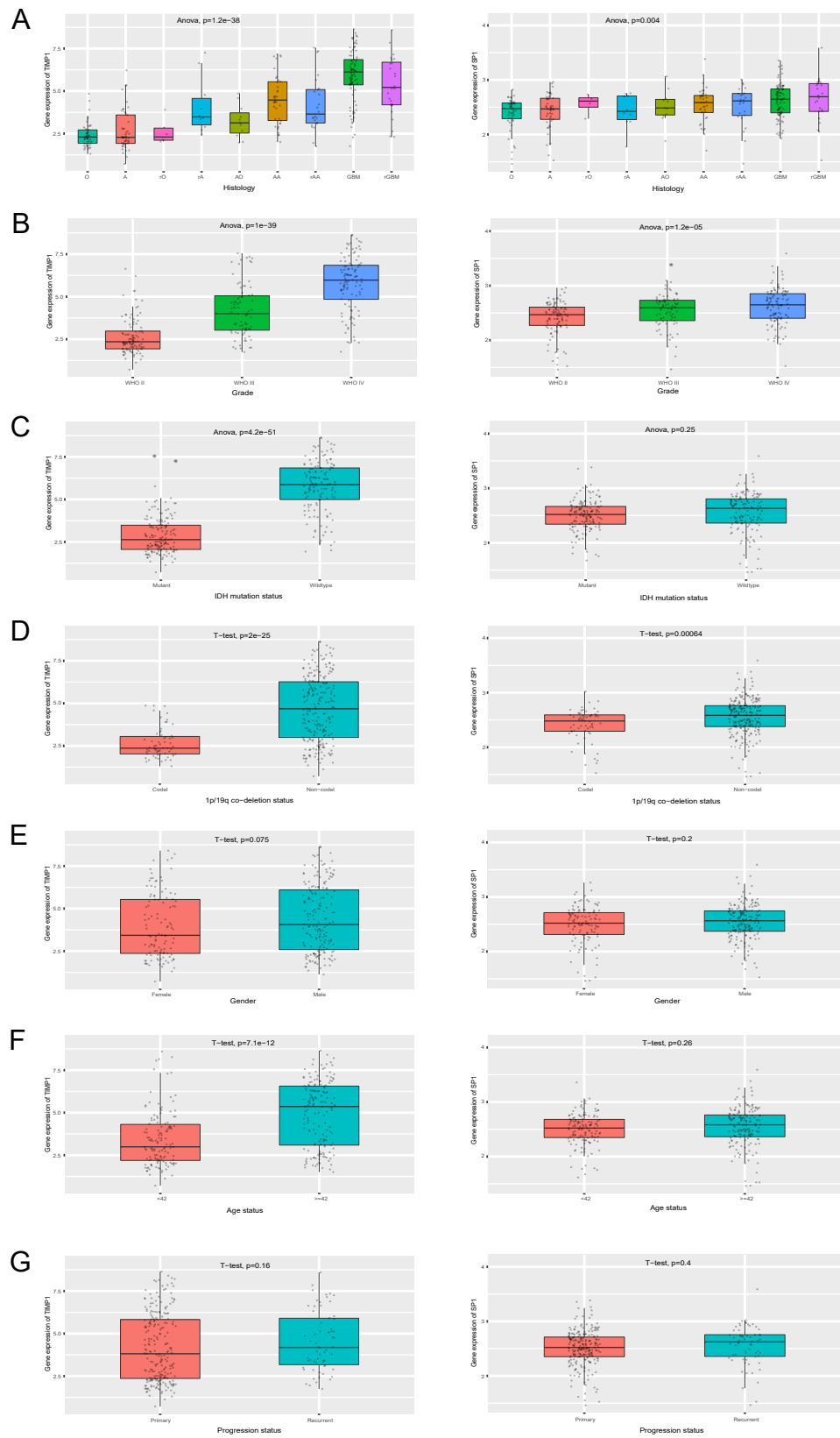

Supplement: Supplementary file 8 — Supplementary Figure S8. [file 41598_2022_14751_MOESM8_ESM.pdf]
