# Supplementary material for: Sp1 induced gene TIMP1 is related to immune cell infiltration in glioblastoma
Source: Sci Rep. 2022 Jul 1;12:11181. doi: 10.1038/s41598-022-14751-4 (PMC9249770; doi:10.1038/s41598-022-14751-4)
Supplement: Supplementary file 6 — Supplementary Figure S6. [file 41598_2022_14751_MOESM6_ESM.pdf]

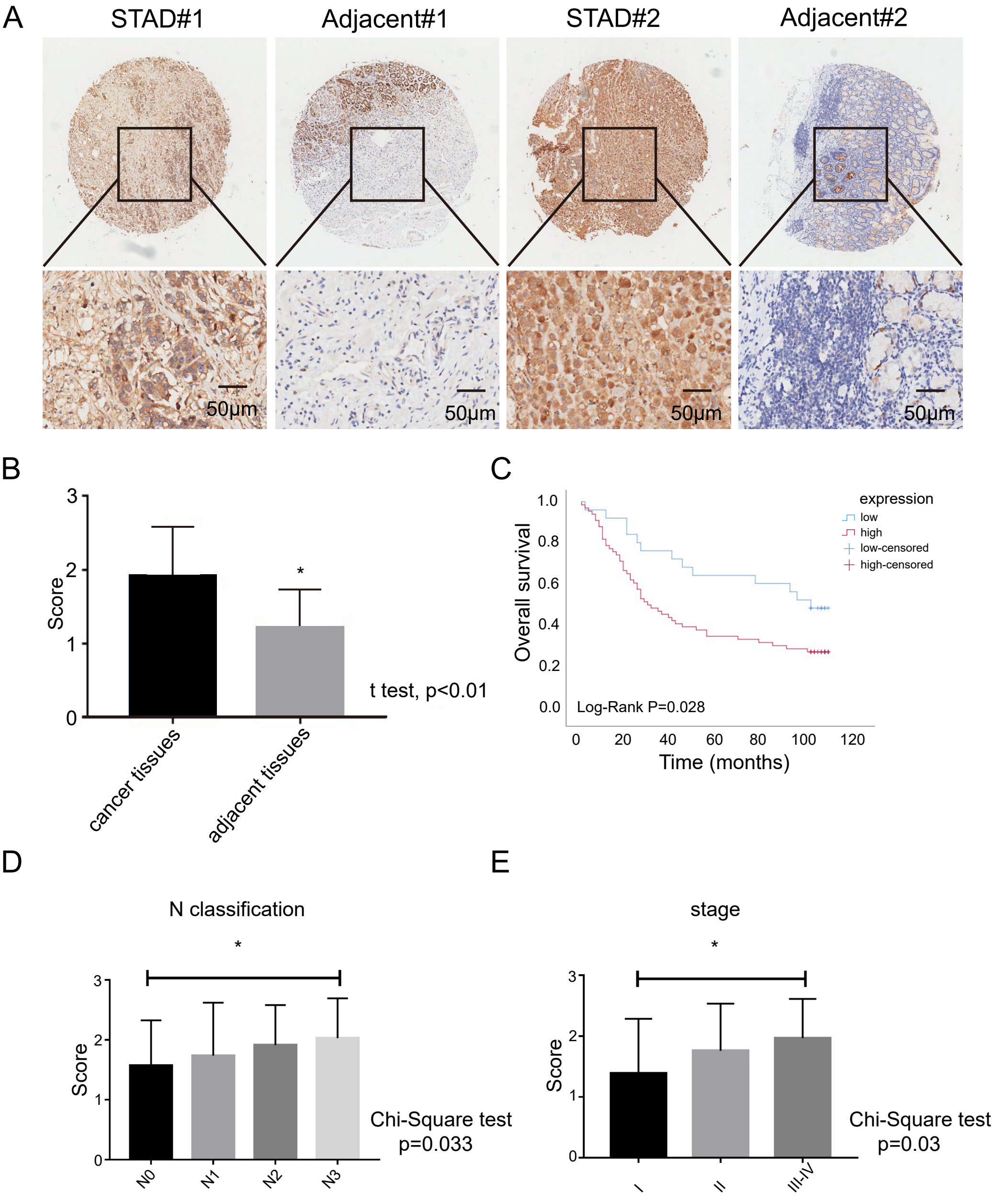

F

Variables in the Equation

variables	Univariate analysis			Multivariate analysis		
	HR	95%CI	p value	HR	95%CI	p value
expression	1.954	1.057-3.614	0.033	1.222	0.644-2.318	0.54
sex	0.868	0.517-1.457	0.591			
age	1.224	0.736-2.035	0.437			
grade	1.906	1.102-3.299	0.021	1.377	0.742-2.41	0.334
T	2.057	1.362-3.108	0.001	1.555	0.943-2.567	0.084
N	1.823	1.416-2.346	<0.001	1.419	0.924-2.179	0.11
TNM	3.273	1.93-5.55	<0.001	1.369	0.523-3.584	0.522
