# Supplementary material for: Sp1 induced gene TIMP1 is related to immune cell infiltration in glioblastoma
Source: Sci Rep. 2022 Jul 1;12:11181. doi: 10.1038/s41598-022-14751-4 (PMC9249770; doi:10.1038/s41598-022-14751-4)
Supplement: Supplementary file 13 — Supplementary Information. [file 41598_2022_14751_MOESM13_ESM.docx]

LN229 in Figure 7 C

The blots of Sp1


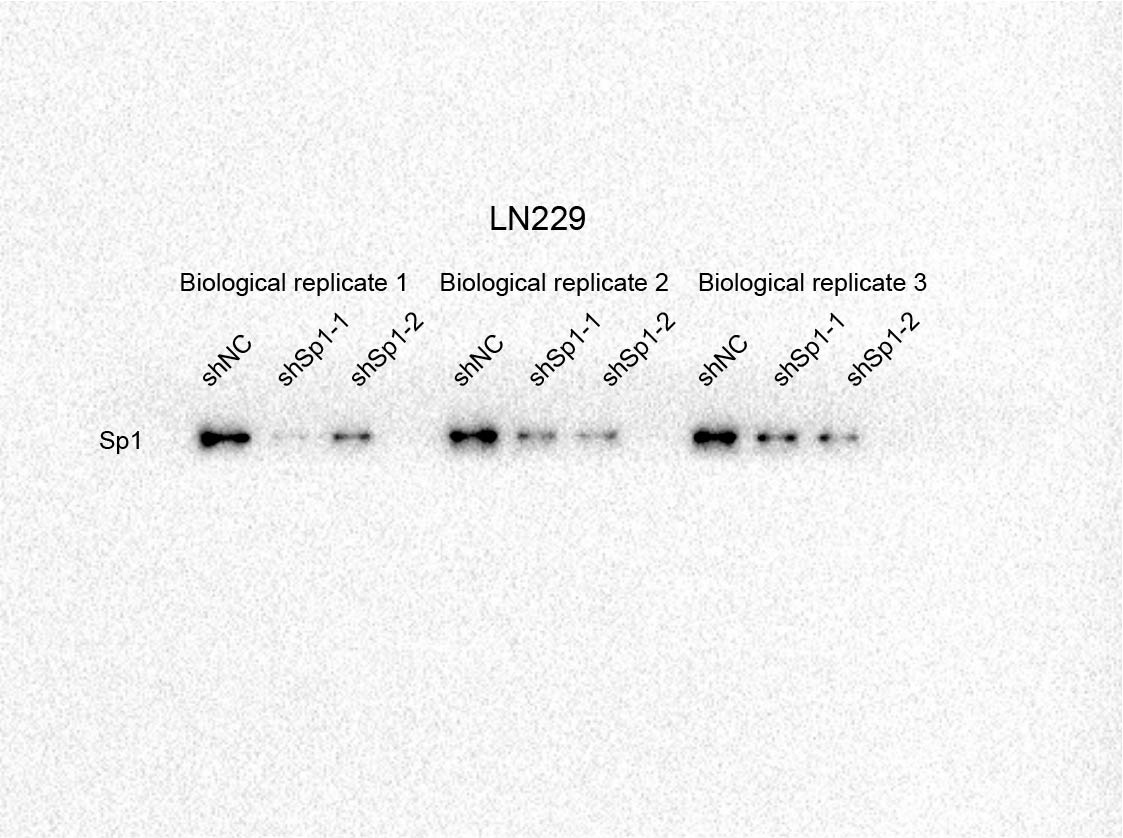


A


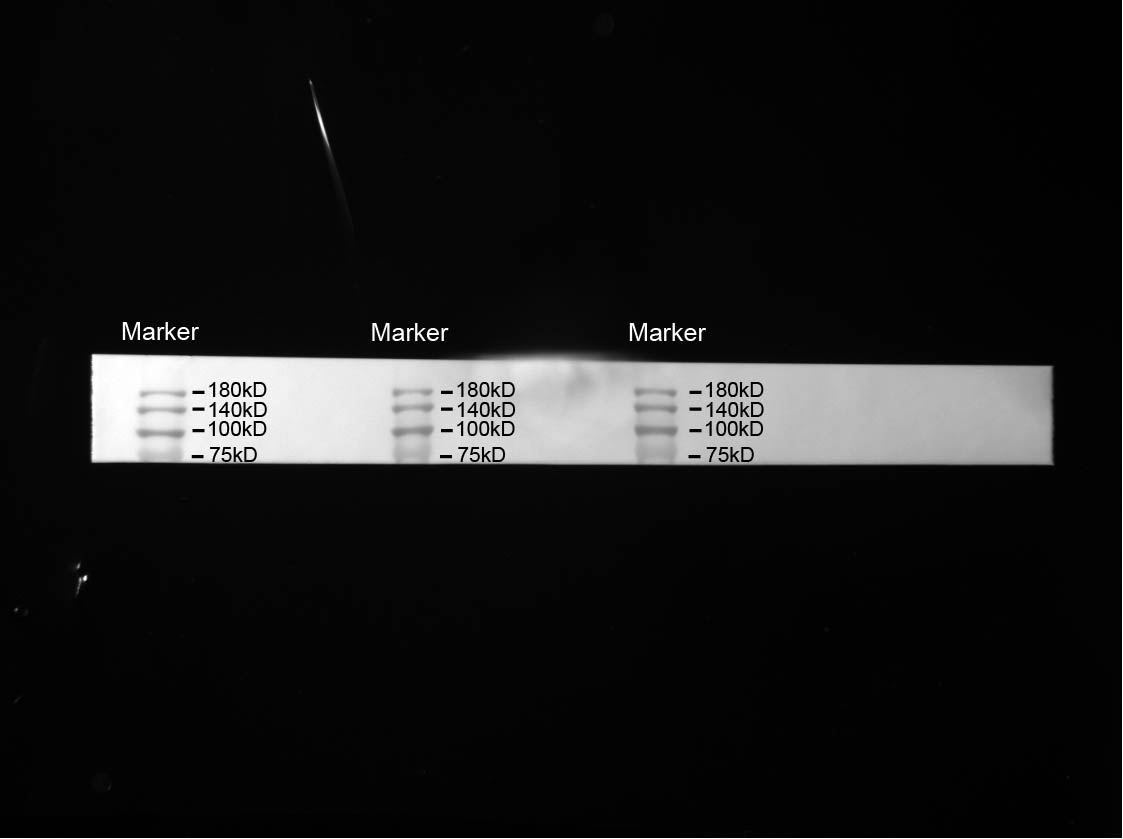


B


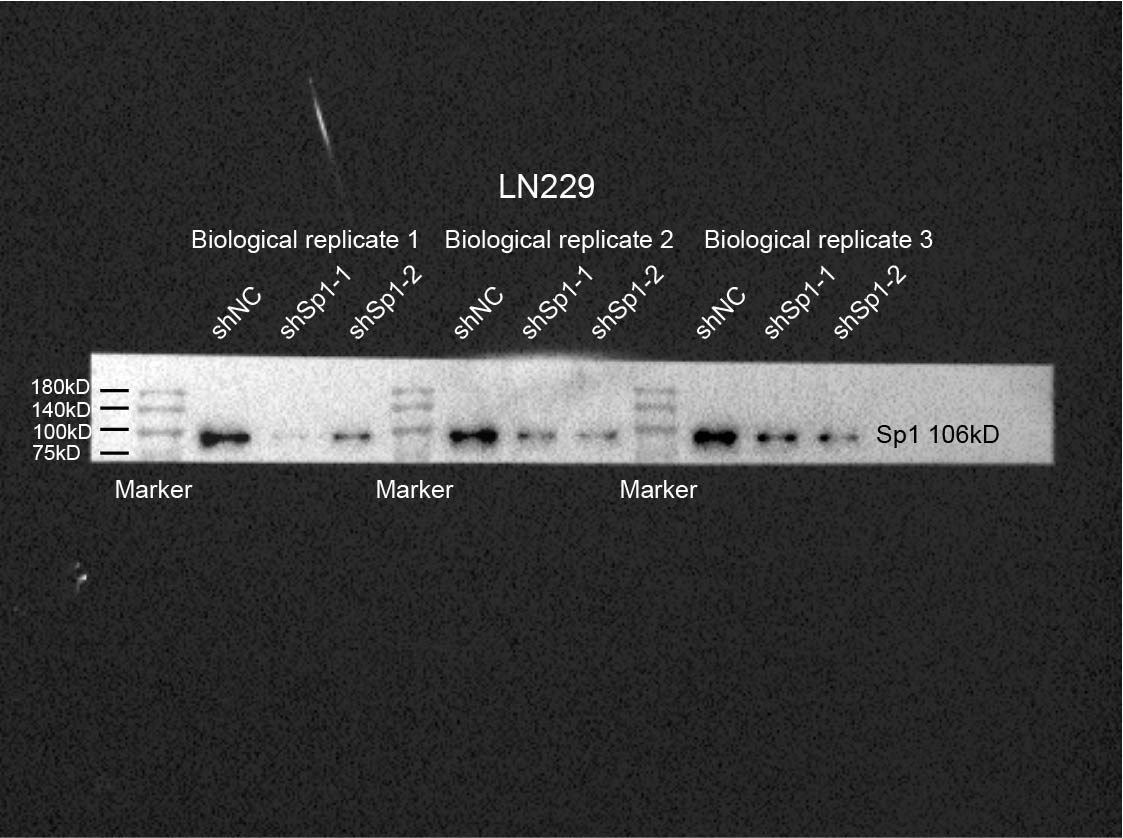


C

Figure A indicates the western blot of Sp1 and its group including shNC, shSP1-1, shSP1-2. n=3 independent experiments. Figure B is the marker image of this western blot. Figure C is the merged image of figure A and figure B.

The blots of ACTB


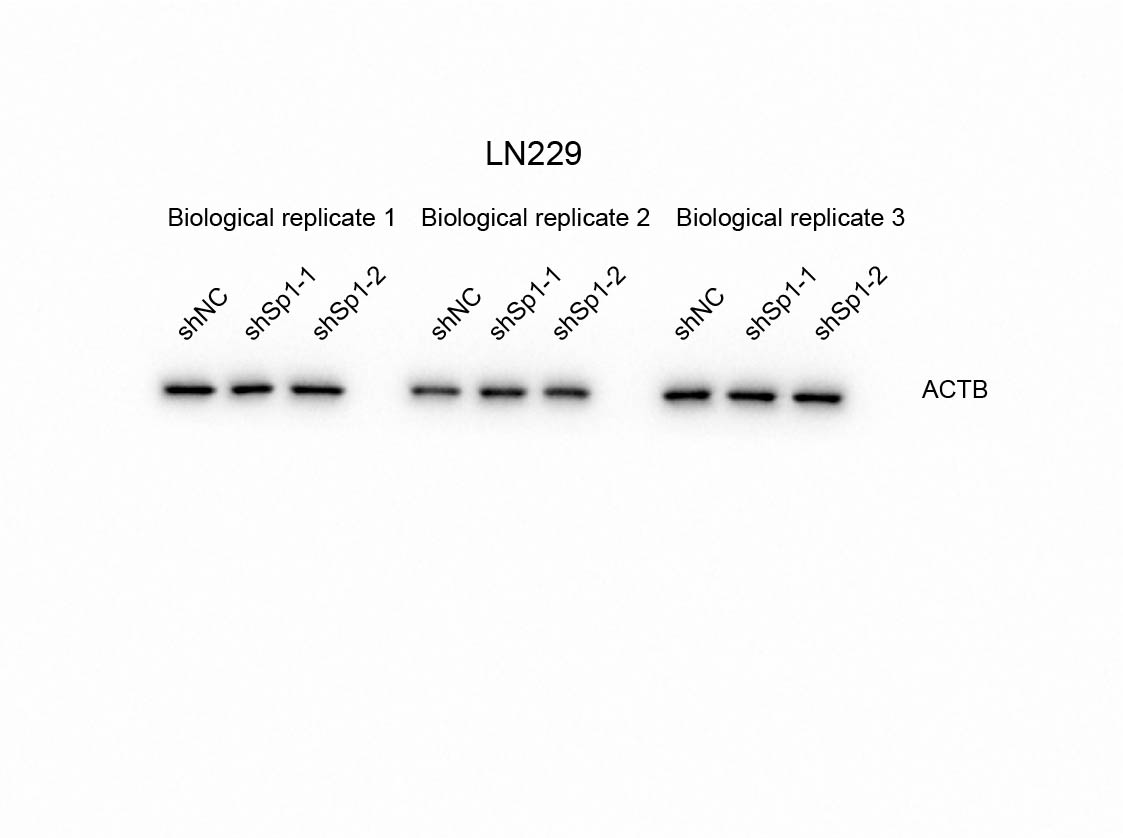


A


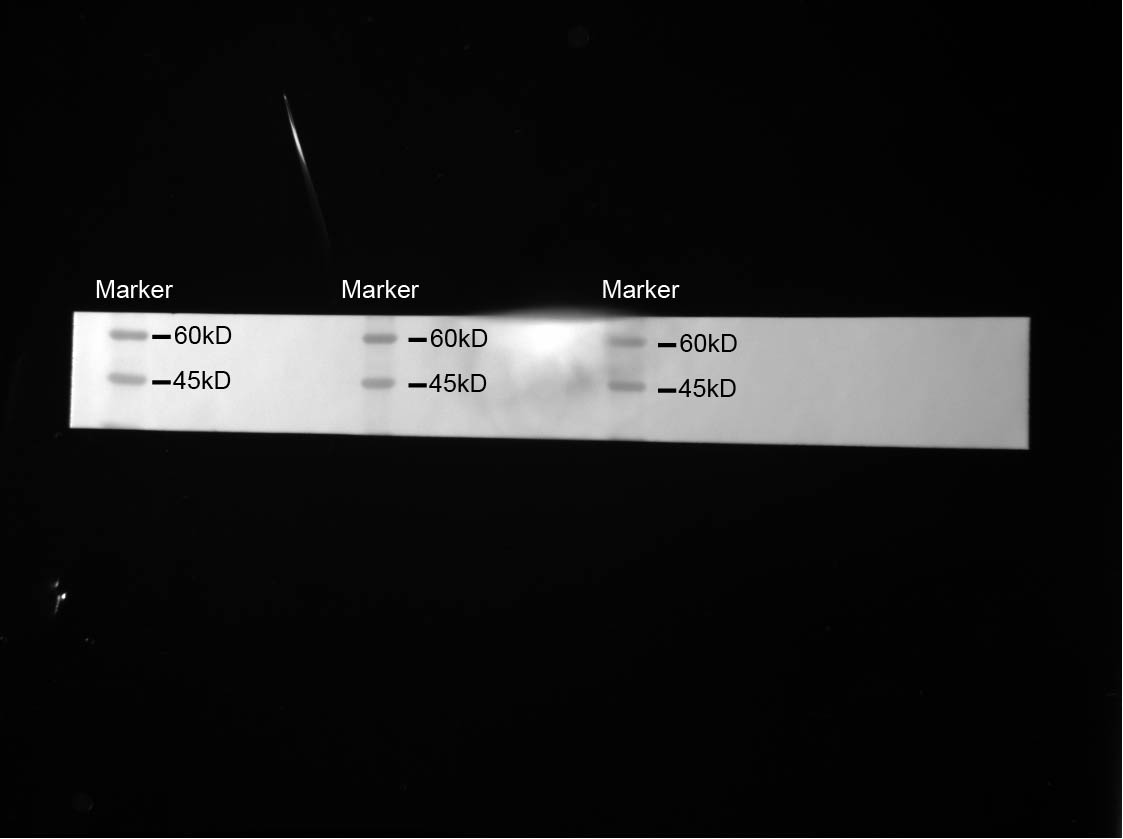


B


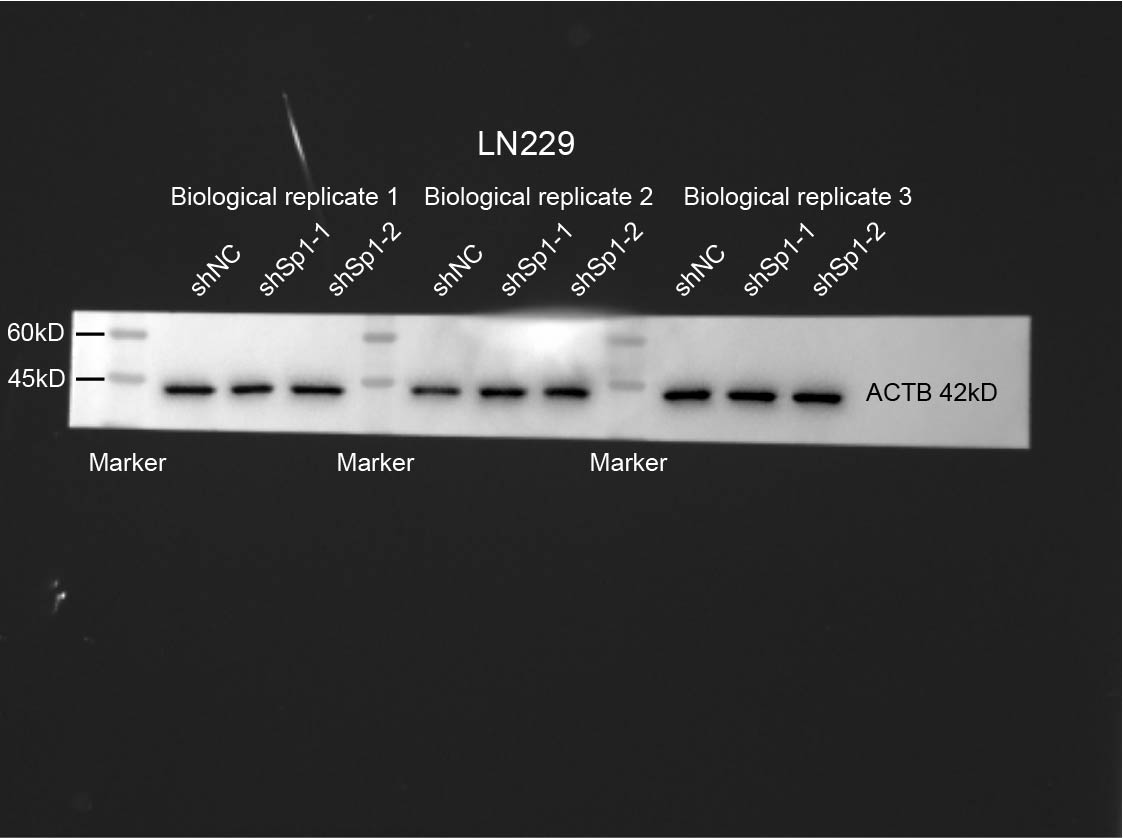


C

Figure A indicates the western blot of ACTB and its group including shNC, shSP1-1, shSP1-2. n=3 independent experiments. Figure B is the marker image of this western blot. Figure C is the merged image of figure A and figure B.

The blots of TIMP1


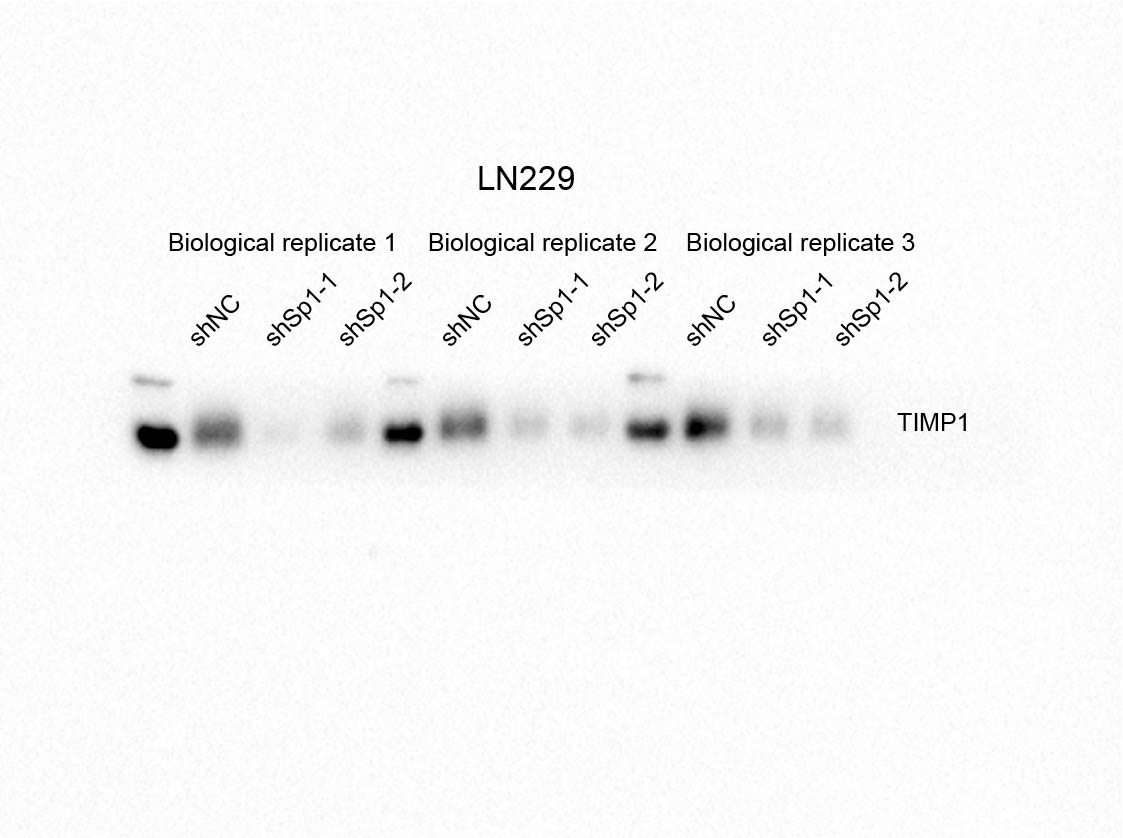


A


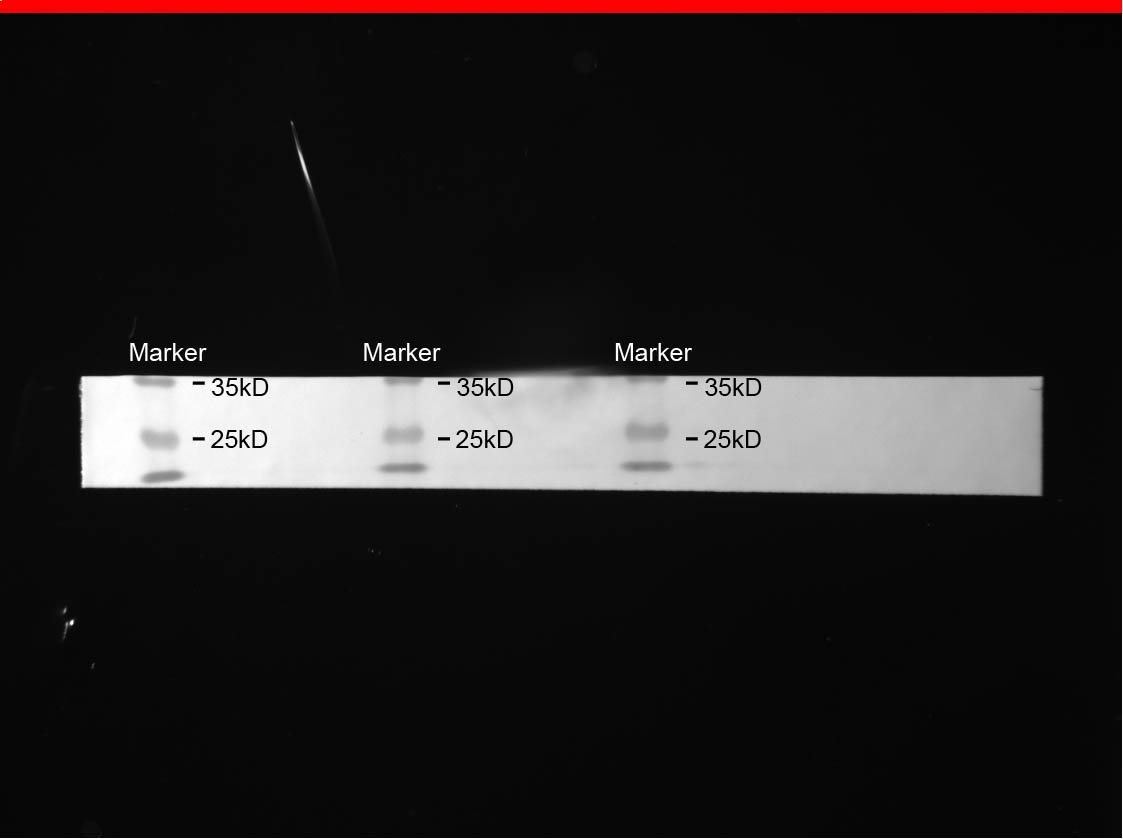


B


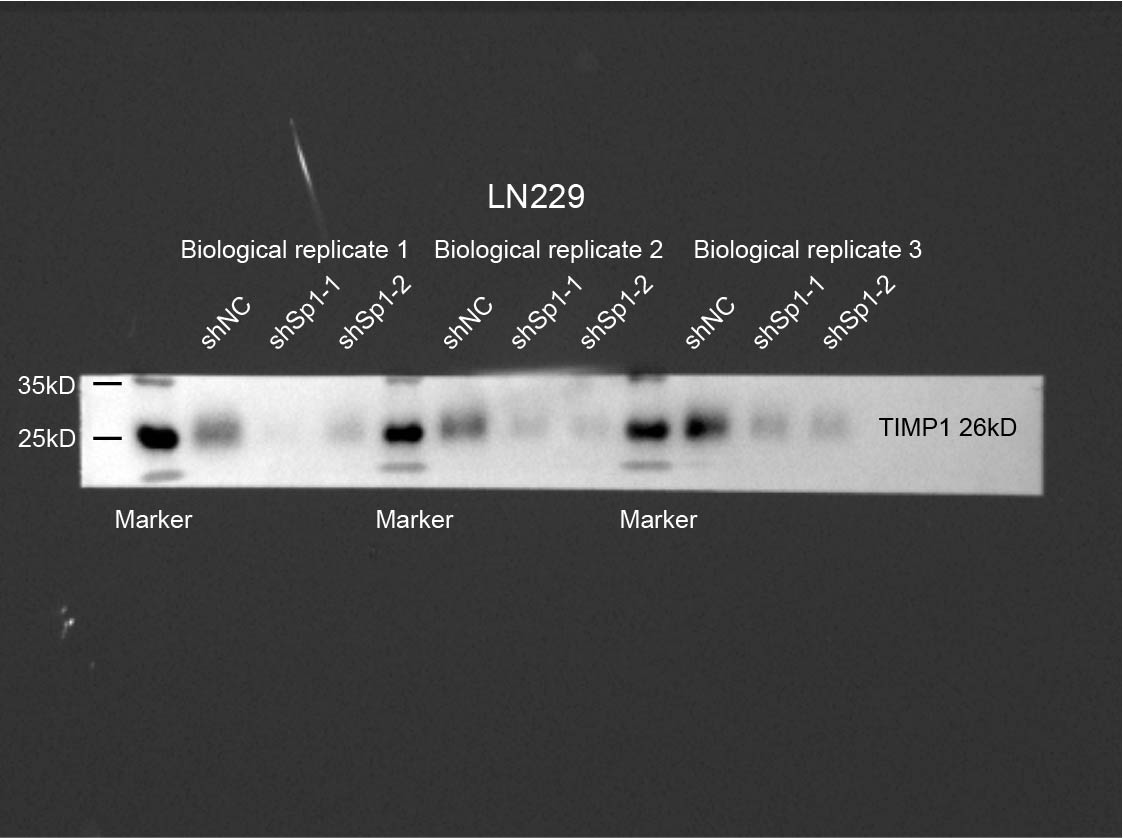


C

Figure A indicates the western blot of TIMP1 and its group including shNC, shSP1-1, shSP1-2. n=3 independent experiments. Figure B is the marker image of this western blot. Figure C is the merged image of figure A and figure B.

U251 in Figure 7 D

The blots of Sp1


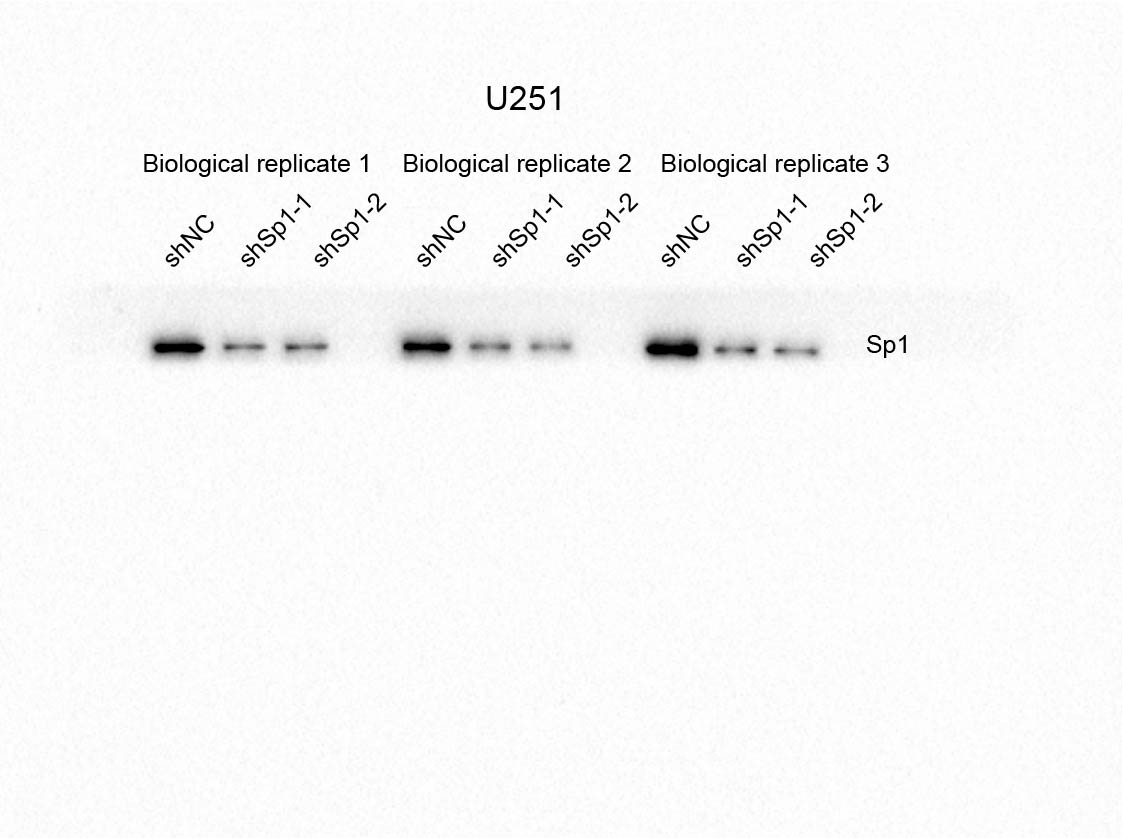


A


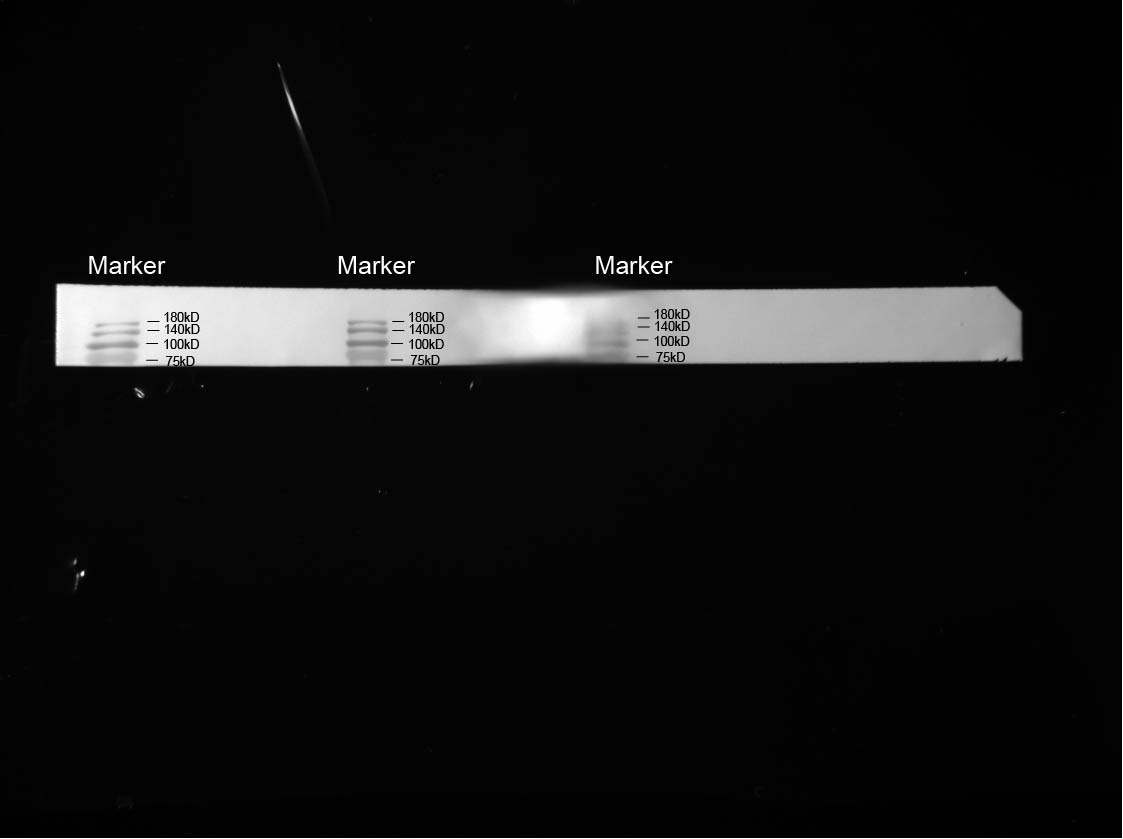


B


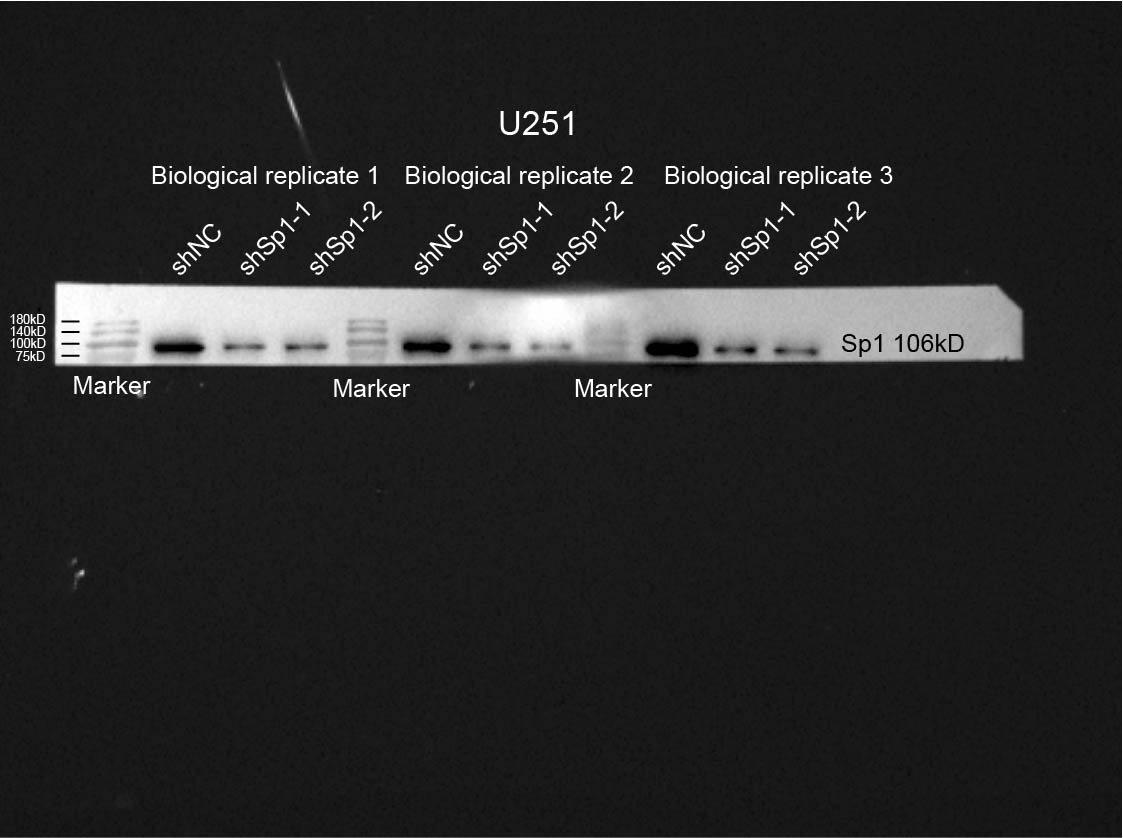


C

Figure A indicates the western blot of Sp1 and its group including shNC, shSP1-1, shSP1-2. n=3 independent experiments. Figure B is the marker image of this western blot. Figure C is the merged image of figure A and figure B.

The blots of ACTB


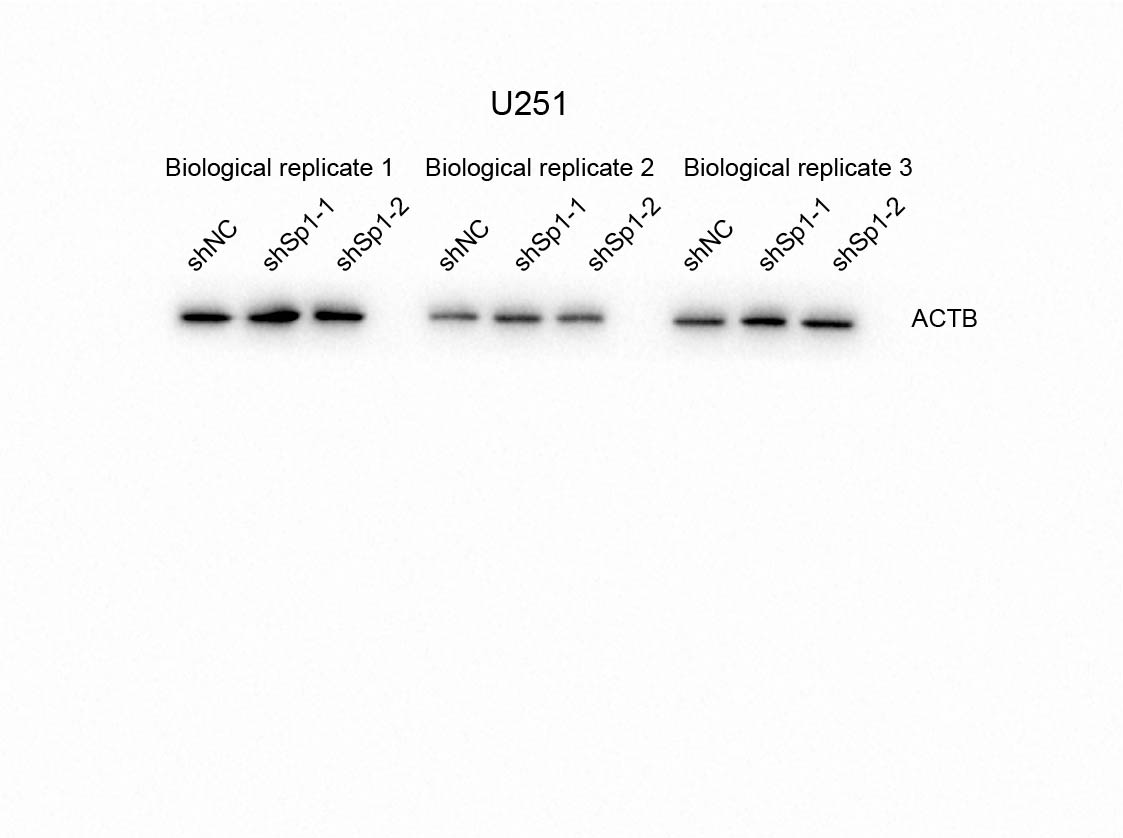


A


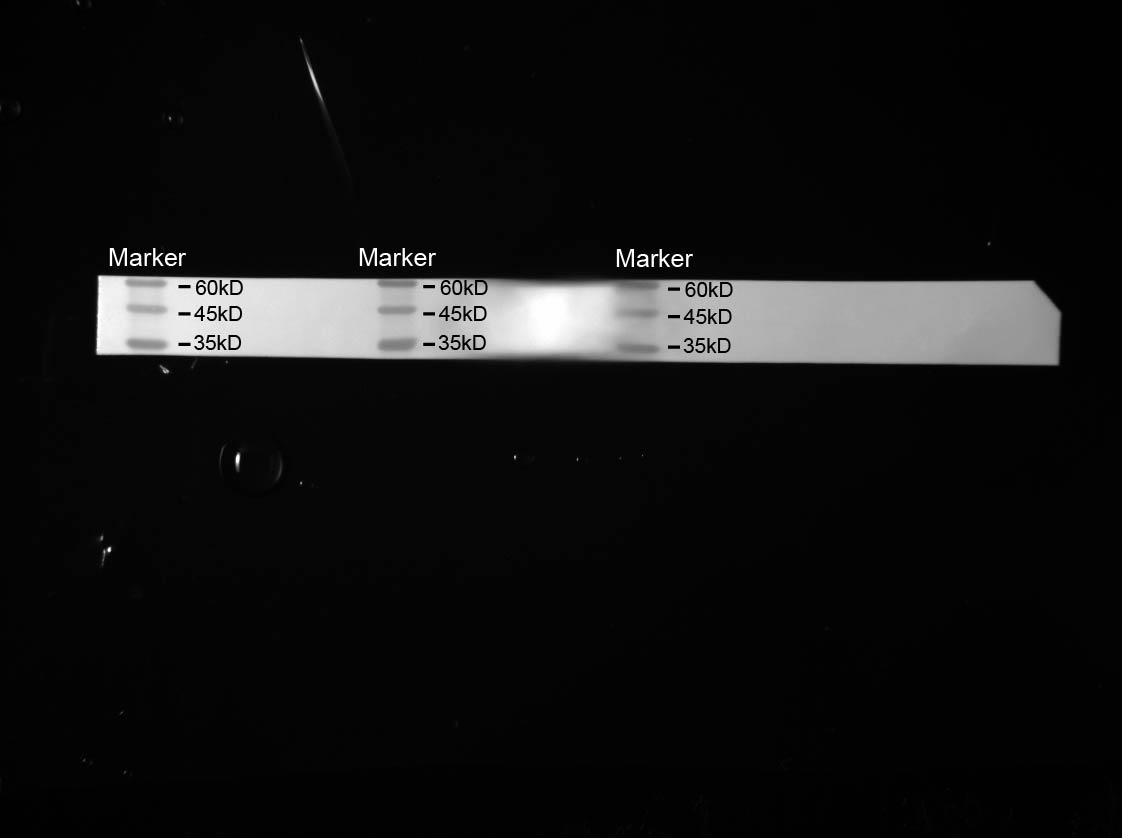


B


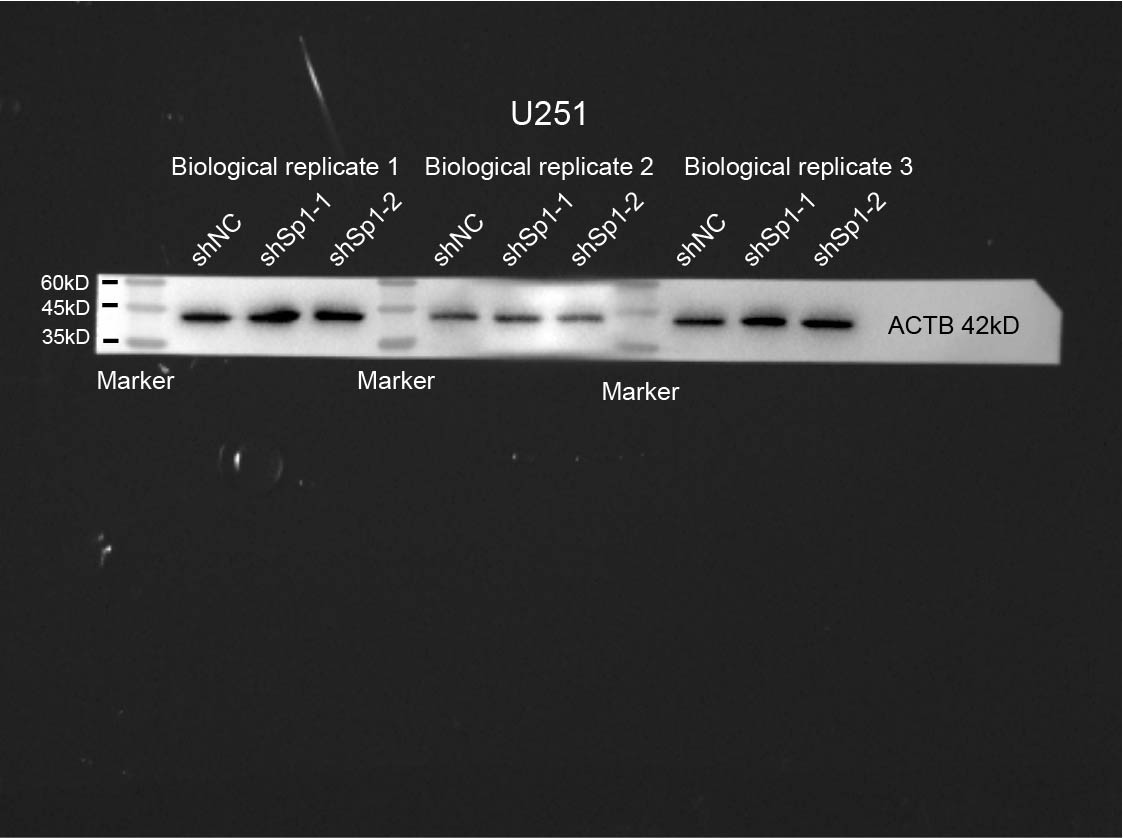


C

Figure A indicates the western blot of ACTB and its group including shNC, shSP1-1, shSP1-2. n=3 independent experiments. Figure B is the marker image of this western blot. Figure C is the merged image of figure A and figure B.

The blots of TIMP1


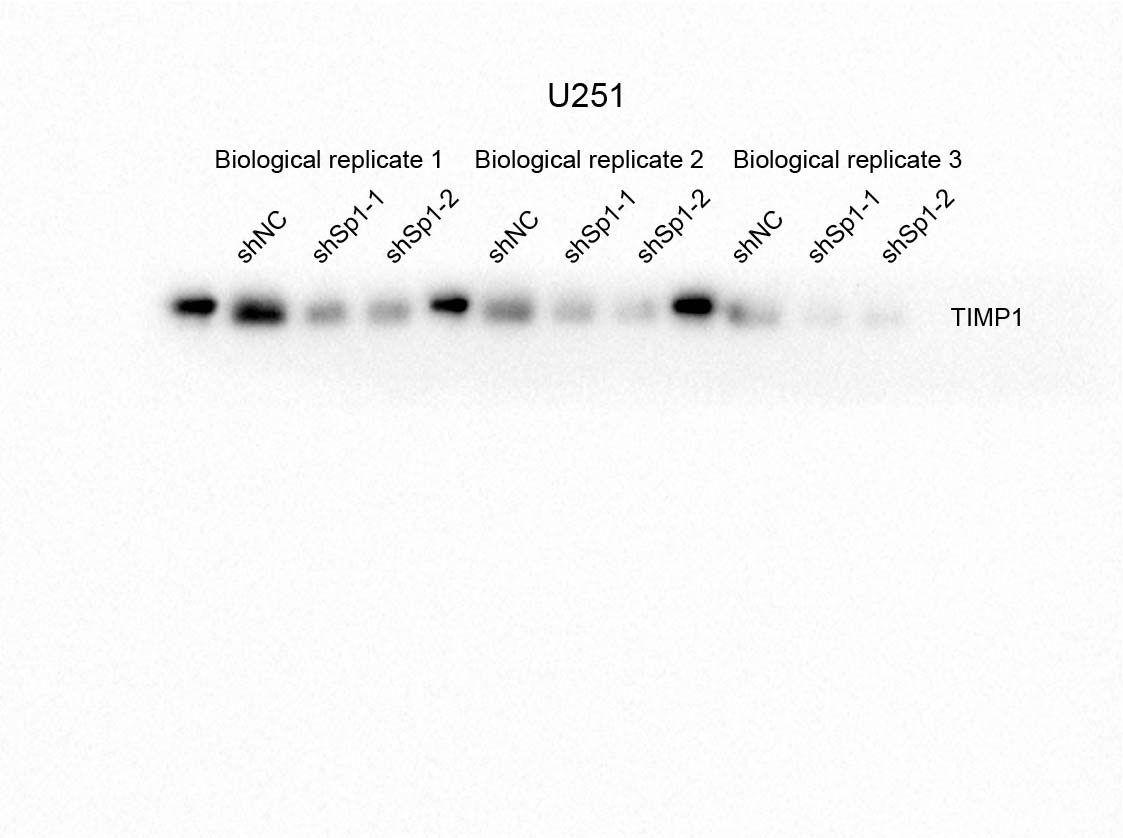


A


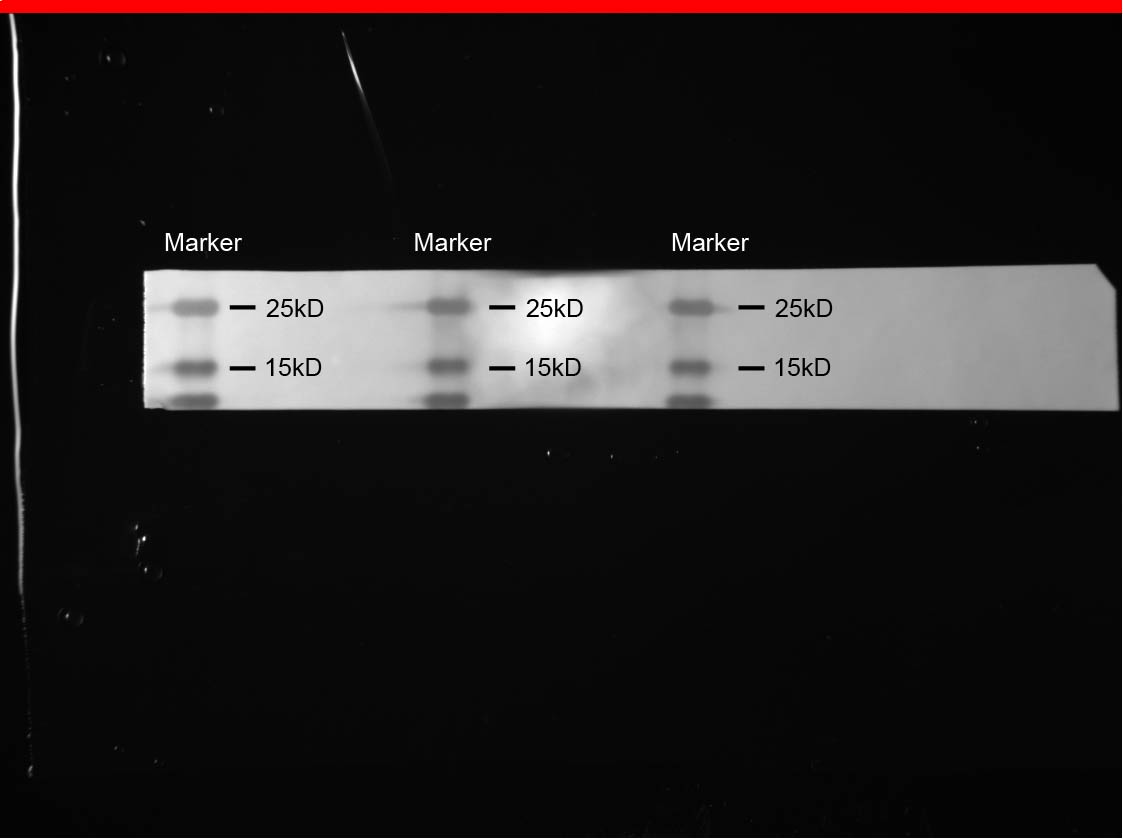


B


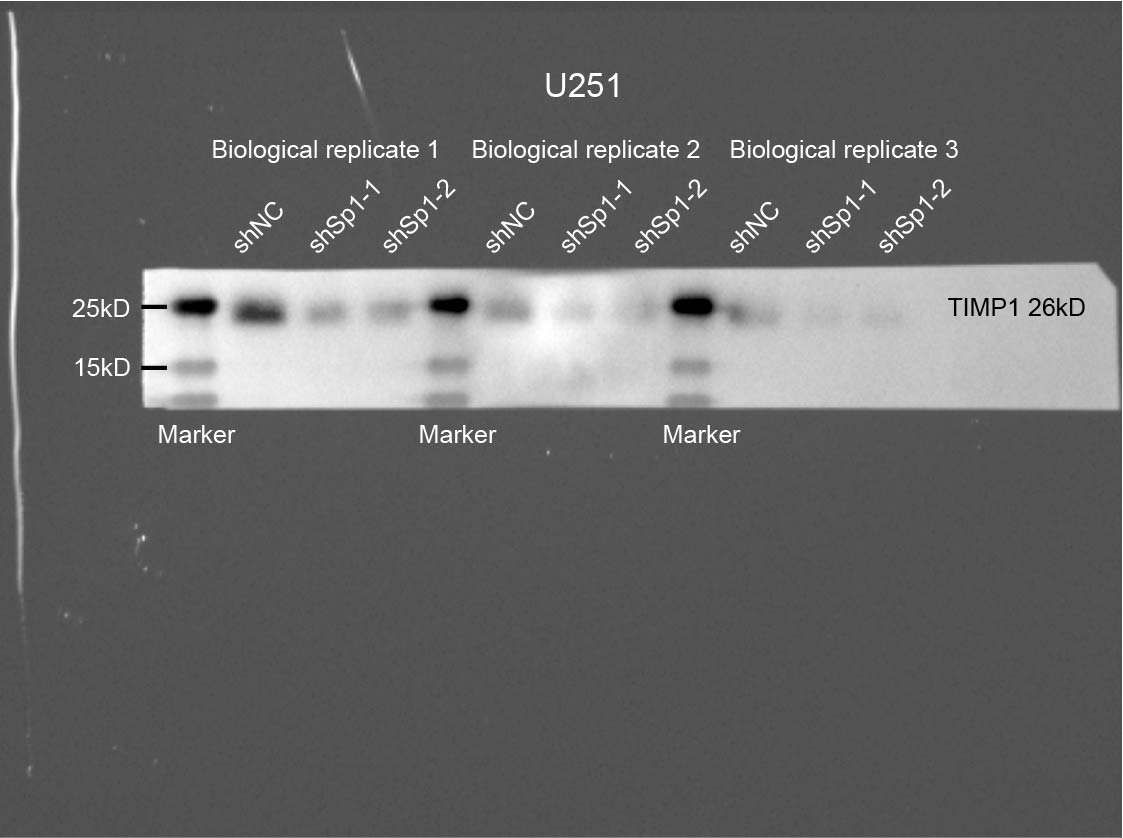


C

Figure A indicates the western blot of TIMP1 and its group including shNC, shSP1-1, shSP1-2. n=3 independent experiments. Figure B is the marker image of this western blot. Figure C is the merged image of figure A and figure B.

The gel in Figure 7G and 7H


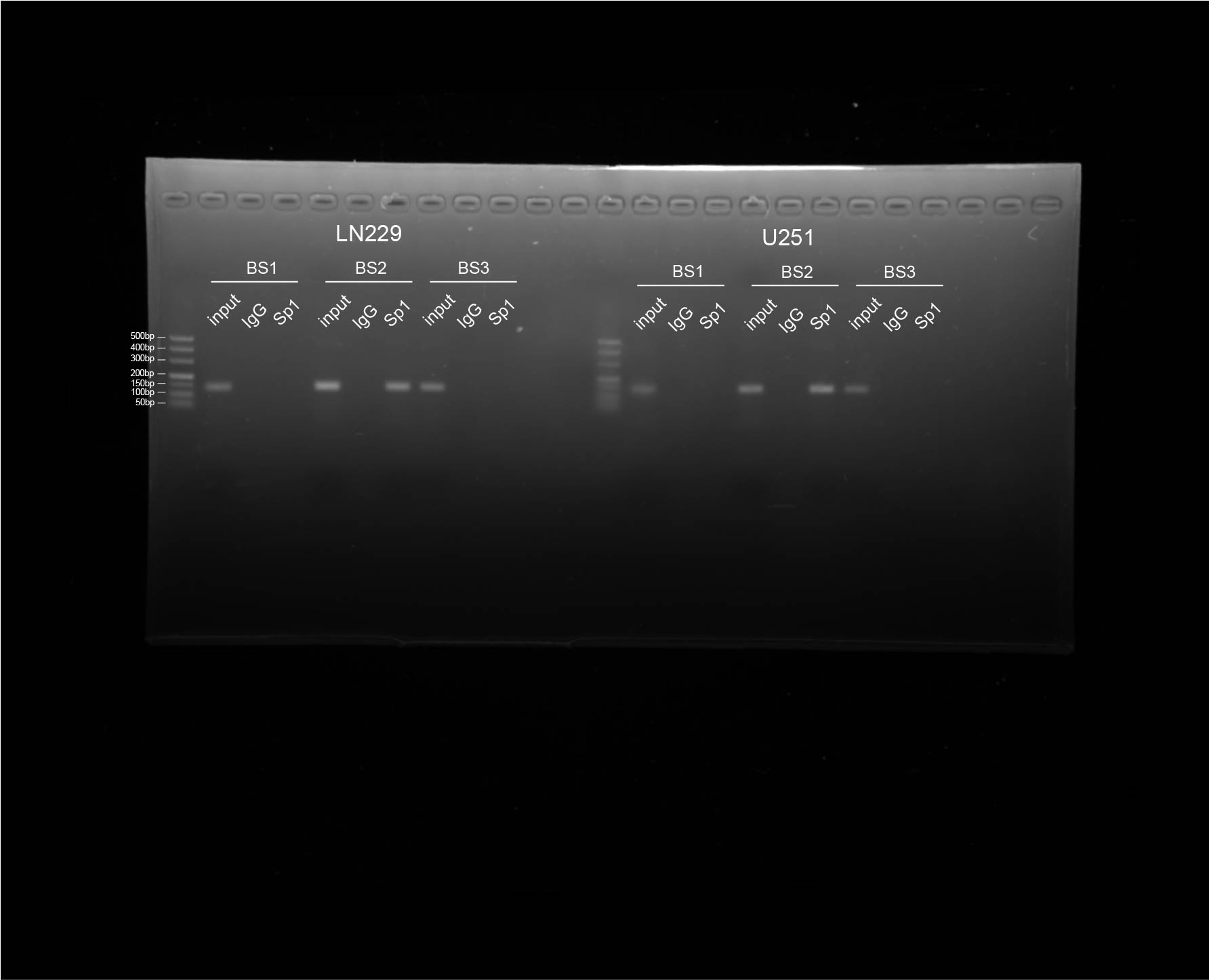


The left pannel is the result of LN229 and the right pannel is the result of U251.
