# Supplementary material for: Sp1 induced gene TIMP1 is related to immune cell infiltration in glioblastoma
Source: Sci Rep. 2022 Jul 1;12:11181. doi: 10.1038/s41598-022-14751-4 (PMC9249770; doi:10.1038/s41598-022-14751-4)
Supplement: Supplementary file 14 — Supplementary Legends. [file 41598_2022_14751_MOESM14_ESM.docx]

**Sp1 Induced Gene TIMP1 is Related to Immune Cell Infiltration in Glioblastoma**

**Lu Liu1+, Shuyao Yang1+, Kefeng Lin1+, Xiaoman Yu2, Jiaqi Meng1, Chao Ma1, Zheng Wu1, Yuchao Hao1, Ning Chen1, Qi Ge1, Wenli Gao1, Xiang Wang1, Eric W.-F. Lam3, Lin Zhang1, Fangcheng Li2*, Bilian Jin1* and Di Jin1***

**Supplementary Materials**

**Table S1.** The correlation between TIMP1 expression and survival for patients at different stages of stomach adenocarcinoma with lymphatic metastasis.

**Table S2.** The correlations between TIMP1 expression and levels of tumor-infiltrating immune cells.

**Table S3.** The correlations between TIMP1 expression and immune marker sets in TIMER.

**Figure S1.** The differential expression levels of TIMP1 in different forms of human cancer. **(A)** The differential TIMP1 expression revealed in the Oncomine database. Red indicates high expression of TIMP1, blue indicates low expression of TIMP1 and number indicates the number of TIMP1 expression cases. **(B)** Levels of TIMP1 expression in different forms of human cancers revealed by TIMER analysis. Notably, red represents tumor sample, blue represents normal sample and purple represents metastatic sample. ** p <0.05，** p <0.01，*** p <0.001*. **(C)** The forest plot of 33 up-regulated genes for prognosis. **(D)** ROC of risk score of TCGA-GBM datasets. **(E)** The boxplot of risk score between different patient statuses. Event 0 indicates the alive status of the patients and event 1 the dead status of the patients in GBM.

**Figure S2.** The correlations between TIMP1 expression and survival via PROGgene. **(A)** Glioblastoma multiforme (GBM), **(B-C)** Brain lower-grade glioma (LGG), **(D)** Kidney clear cell carcinoma (KIRC), **(E)** Kidney renal clear cell carcinoma (KIRC), **(F)** Kidney renal papillary cell carcinoma (KIRP), **(G-H)** Colon adenocarcinoma/Rectum adenocarcinoma Esophageal carcinoma (COAD/READ), **(I)** Uveal melanoma (UVM), **(J-K)** Stomach adenocarcinoma (STAD), **(L)** Head and neck squamous cell carcinoma (HNSC). PVAL for P value. Green lines indicate low expression of TIMP1 and red lines indicate high expression of TIMP1. The groups withlow and high expression of TIMP1 were stratified according to their median expression levels of TIMP1. Data were analyzed by log-rank test.

**Figure S3.** The correlations between TIMP1 and overall survival (OS)/ post progression survival (PPS)/ progression free survival (PFS) via Kaplan-Meier Plotter. **(A-B)** Gastric cancer, **(C-D)** Lung adenocarcinoma (LUAD), **(E-F)** Breast cancer, **(G-H)** Ovarian cancer. **(H-I)** Glioblastoma (GBM), **(J-K)** Brain lower-grade glioma (LGG). Black lines indicate low expression of TIMP1 and red lines indicate high expression of TIMP1. The groups with low and high expression of TIMP1 were stratified according to the median expression level of TIMP1. Data were analyzed by log-rank test. **(A-H)** were analyzed through Kaplan-Meier Plotter web, **(H-I)** were analyzed through TCGA-GBM microassay data, and **(J-K)** were analyzed through TCGA-LGG RNA-seq data.

**Figure S4.** The correlations between TIMP1 expression and different forms of immune cell infiltrates in different cancer types. **(A)** TIMP1 expression is significantly negatively related to tumor purity and has a positive correlation with infiltrating levels of CD8+ T cells, CD4+ T cells, macrophages, neutrophils, and dendritic cells in colon adenocarcinoma (COAD). **(B)** TIMP1 expression is significantly negatively related to tumor purity and has a positive correlation with infiltrating levels of B cells, CD8+ T cells, CD4+ T cells, macrophages, neutrophils, and dendritic cells in head and neck squamous cell carcinoma (HNSC). **(C)** TIMP1 expression is significantly negatively related to tumor purity and has a positive correlation with infiltrating levels of CD4+ T cells in kidney renal clear cell carcinoma (KIRC). **(D)** TIMP1 expression is significantly negatively related to tumor purity and has a positive correlation with infiltrating levels of B cells, CD8+ T cells, CD4+ T cells, macrophages, neutrophils, and dendritic cells in brain lower-grade glioma (LGG). **(E)** TIMP1 expression is significantly negatively related to tumor purity and has a positive correlation with infiltrating levels of B cells, CD4+ T cells, macrophages, neutrophils, and dendritic cells in lung adenocarcinoma (LUAD). **(F)** TIMP1 expression is significantly negatively related to tumor purity and has a positive correlation with infiltrating levels of CD4+ T cells, macrophages, neutrophils, and dendritic cells in rectum adenocarcinoma (READ). **(G)** TIMP1 expression is significantly negatively related to tumor purity and has a positive correlation with infiltrating levels of CD8+ T cells, macrophages, neutrophils, and dendritic cells in the stomach adenocarcinoma (STAD).

**Figure S5.** The associations between TIMP1 expression and immune marker sets on **(A)** CD8+ T cells, T cells, B cells, monocytes, **(B)** TAMs, and macrophages in STAD，LGG and GBM. Markers include CD8A, CD8B of CD8+ T cells, CD2, CD3E, CD3D of T cells, CD19, CD79A of B cells, CD115, CD86 of monocytes, CCL2, CD68, IL10 of TAMs (tumor-associated macrophages), COX2, INOS, IRF5 of M1 macrophages, CD163, MS4A4A, and VSIG4 of M2 macrophages.

**Figure S6.** Up-regulated TIMP1 expression is associated with poor outcome of STAD. **(A)** Representative IHC images of TIMP1 staining in STAD tumors and adjacent tissues (magnification, 3× and 20×). Blue color indicates staining for nuclei and brown color indicates staining for TIMP1 protein.**(B)** IHC scores of TIMP1 staining in 86 pairs of STAD tissues. Data were analyzed by *t* test. **(C)** Kaplan-Meier analysis of overall survival of 94 STAD patients. The low expression and high expression of TIMP1 were grouped by the IHC total score. Data were analyzed by log-rank test. **(D)** IHC scores of TIMP1 staining in STAD tissues with different stages and the p value between N0 and N1/N2/N3 is 0.033. Data were analyzed by Chi-Square test. **(E)** IHC scores of TIMP1 staining in STAD tissues with different pathological grades and the p value between I/II and III/IV is 0.03. Data were analyzed by Chi-Square test. **(F)** Univariate and multivariate regression analyses of TIMP1 for overall survival in STAD patients.

**Figure S7.** The differential expressions, survival and correlation analysis of predictive transcriptional factors. **(A)** The expression level of Sp1 in glioma cells compared with normal brain tissues from TCGA. The red box indicates tumor samples and the black box indicates normal samples. **(B)** The correlation of overall survival with Sp1expression from CGGA. The groups between low and high expression of Sp1 were stratified according to the median expression level of Sp1. Data were analyzed by log-rank test. **(C)** The correlation between TIMP1 and Sp1 expression from CGGA. **(D)** Upper panel, Sp1 expression is significantly positively related to tumor purity and has a negative correlation with infiltrating levels of macrophage cells in GBM; Lower panel, Sp1 expression is significantly positively related with tumor purity and has a positive correlation with infiltrating levels of B cells and macrophage cells in LGG. **(E)** The correlation of overall survival with TIMP1/Sp1 expression in GBM patients. Left panel shows patients with no chemotherapy and right panel indicates patients with chemotherapy. The groups between TIMP-/Sp1- and TIMP1+/Sp1+ were stratified according to the median expression levels of TIMP1 and Sp1. Data were analyzed by log-rank test. **(F)** The correlation of overall survival with TIMP1/Sp1 expression in GBM patients. Left panel shows patients with no radiotherapy and right panel indicates patients with radiotherapy. The groups between TIMP-/Sp1- and TIMP1+/Sp1+ were stratified according to the median expression level of TIMP1 and Sp1. Data were analyzed by log-rank test. **(E-F)** The data were analyzed through TCGA-GBM microassay data.

**Figure S8.** The clinical characterization of Sp1 and TIMP1 of GBM in CGGA database. The expression of Sp1 and TIMP1 in histology **(A)**, WHO grade **(B)**, IDH mutation status **(C)**, 1p/19q co-deletion status **(D)**, gender **(E)**, age **(F)** and progression **(G)**. O, oligodendroglioma; A, astrocytoma; rO, recurrence of oligodendroglioma; rA, recurrence of astrocytoma; AO, anaplastic oligodendro; AA, anaplastic astrocytoma; rAA, recurrence of anaplastic astrocytoma; GBM, glioblastoma; rGBM, recurrence of GBM.
